# Hybrid Cell Membrane‐Engineered Nanocarrier for Triple‐Action Strategy to Address *Pseudomonas aeruginosa* Infection

**DOI:** 10.1002/advs.202411261

**Published:** 2024-12-25

**Authors:** Shunhao Zhang, Tianyu Chen, Weitong Lu, Yunfeng Lin, Mi Zhou, Xiaoxiao Cai

**Affiliations:** ^1^ State Key Laboratory of Oral Diseases National Center for Stomatology National Clinical Research Center for Oral Diseases West China Hospital of Stomatology Sichuan University Chengdu Sichuan 610041 China; ^2^ Sichuan Provincial Engineering Research Center of Oral Biomaterials Chengdu Sichuan 610041 China; ^3^ National Center for Translational Medicine Shanghai Jiao Tong University Shanghai 200240 China

**Keywords:** biofilm, cell membrane coating, drug delivery, framework nucleic acid, inflammation targeting, *Pseudomonas aeruginosa* (*P. aeruginosa*), ventilator‐associated pneumonia (VAP)

## Abstract

Bacterial infections resistant to antimicrobial treatments, particularly those caused by *Pseudomonas aeruginosa* (*P. aeruginosa*), frequently lead to elevated mortality rates. Tackling this resistance using therapeutic combinations with varied mechanisms has shown considerable promise. In this study, a bioinspired nanocarrier is successfully designed and engineered for targeted antibiotic delivery and toxin/bacteria clearance. This is achieved by encapsulating antibiotic‐loaded framework nucleic acids with hybrid cell membranes acquired from neutrophils and platelets. By coating the hybrid membrane outside the shell, nanocarriers are endowed with the function of neutrophil‐like chemotaxis and platelet‐like bacteria adhesion to achieve the first stage of inflammation targeting. Based on the specific binding of bacteria toxin to the hybrid membrane, the release of antibiotic‐loaded framework nucleic acids is triggered by toxin‐mediated membrane lysis to fulfill the second stage of toxin neutralization and bacteria killing. Meanwhile, the immunomodulation potential of framework nucleic acids enables nanocarriers to accomplish the third stage of reversing the immunosuppressive microenvironment. In mouse models of acute and chronic *P. aeruginosa* pneumonia, the nanocarriers can reduce bacterial burden at a low dosage and decrease mortality with negligible toxicity. In sum, these findings have illustrated the remarkable capability of nanocarriers in treating recalcitrant bacterial infections.

## Graphical abstract

This study presents a bioinspired nanocarrier designed for targeted antibiotic delivery against *Pseudomonas aeruginosa* infections. Engineered with hybrid cell membranes, the nanocarriers exhibit neutrophil‐like chemotaxis and platelet‐like bacteria adhesion, facilitating effective toxin neutralization and bacterial clearance. In mouse models, they significantly reduce bacterial burden and mortality with minimal toxicity, showcasing their potential for treating resistant infections.

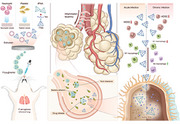

## 1 Introduction

Ventilator‐associated pneumonia (VAP) is a severe and potentially life‐threatening infection that occurs within 48 h after the initiation of mechanical ventilation in intensive care unit (ICU) settings. VAP is commonly seen among hospital patients who require intubation, with a prevalence of ≈10–25%, and results in high mortality rates and prolonged periods of ventilation.^[^ ^1^, ^2^ ^]^ The emergence of antimicrobial‐resistant pathogens, such as *Pseudomonas aeruginosa* (*P. aeruginosa*), has become a growing concern due to the abuse of antibiotics in the clinical treatment of VAP.^[^ ^3^, ^4^, ^5^ ^]^ Biofilm formation stands out as a leading mechanism contributing to the development of antimicrobial resistance in bacterial infections. Biofilms are protective structures formed by bacteria, which encase themselves within the extracellular polymeric substances (EPS) to impede antibiotic penetration.^[^ ^6^, ^7^ ^]^ *P. aeruginosa* biofilms release hemolytic toxins known as pore‐forming toxins (PFTs) into blood, which not only cause pore formation on cell membranes and alter membrane permeability^[^ ^8^, ^9^ ^]^ but also disrupt various immune responses of the host. In particular, they hinder macrophage activation and recruit myeloid‐derived suppressor cells (MDSCs) to lesion sites, creating an immunosuppressive microenvironment that impairs pathogen clearance.^[^ ^10^, ^11^ ^]^ Patients experiencing an acute‐on‐chronic exacerbation due to *P. aeruginosa* infection often require dual coverage with two antibiotics from different classes for a duration of two weeks. This prolonged antibiotic exposure significantly increases the risk of colonization by antimicrobial‐resistant bacteria.^[^ ^12^ ^]^ The combination of antibiotic resistance and suppressed immune function poses considerable challenges to effectively treating *P. aeruginosa*‐induced lung infections.

Over the past few years, tetrahedral framework nucleic acids (tFNAs) have emerged as a breakthrough innovation characterized by their excellent biocompatibility and permeation ability at both cellular and tissue levels, enabling them to transport a wide range of antibacterial drugs efficiently, including antibiotics, antimicrobial peptides, and antisense peptide nucleic acids.^[^ ^13^, ^14^, ^15^, ^16^ ^]^ Moreover, relevant studies have demonstrated the ability of tFNAs to regulate T‐cell differentiation and macrophage polarization, thereby showcasing their potential for immunomodulation.^[^ ^17^, ^18^, ^19^ ^]^ With their exceptional cargo‐carrying capabilities and immunomodulatory properties, tFNAs hold great promise as prospective candidates for the treatment of recalcitrant bacterial infections.

**Figure 1:**
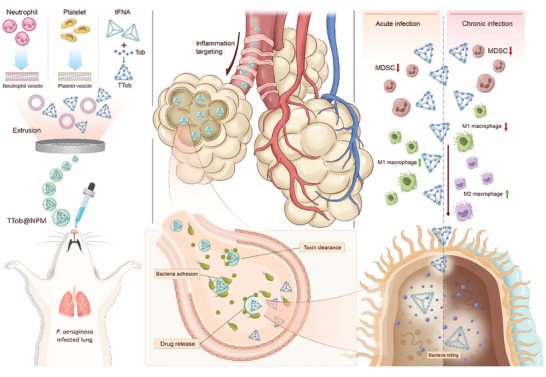
Schematic illustration of the synthesis and function of TTob@NPM. The nanocarrier (TTob@NPM) design includes a complex structure consisting of a tetrahedral framework nucleic acids (tFNAs) loaded with tobramycin (Tob), referred to as TTob. This complex is further coated with a hybrid membrane derived from neutrophil vesicles (NVs) and platelet vesicles (PVs), resulting in a neutrophil‐platelet hybrid membrane (NPM). The incorporation of both neutrophil and platelet membrane characteristics allows the nanocarrier to mimic the lipid membranes and functions of these original cells. The NVs coating provides the nanocarriers with the ability to target inflammation and neutralize toxins. On the other hand, the PVs coating enhances the nanocarriers’ binding capacity to bacteria. This dual coating strategy combines the benefits of both cell types, enabling the nanocarriers to effectively combat *P. aeruginosa* infection. Furthermore, the release of TTob is triggered by toxin‐mediated membrane lysis. This mechanism ensures that TTob is specifically released in the presence of toxins. TTob effectively eliminates bacteria, while the immunomodulation potential of tFNAs plays a crucial role in coordinating macrophage polarization and recruitment of myeloid‐derived suppressor cells (MDSCs) in acute and chronic *P. aeruginosa* infection.

From a therapeutic perspective, the most desirable strategy would involve the concurrent elimination of both toxins and toxin‐producing bacteria to maximize the effectiveness of treatment. Nevertheless, a significant challenge arises from the substantial differences in physicochemical structures and biological targets between toxins and bacteria. These disparities pose a formidable obstacle to achieving simultaneous eradication of both entities. For example, PFTs primarily target neutrophils, whereas bacteria may interact with other cell types, such as platelets.^[^ ^20^, ^21^ ^]^ To address this discrepancy, a bioinspired nanocarrier called TTob@NPM has been developed, which is capable of both delivering antibiotics and clearing toxins/bacteria at the same time. The nanocarrier consists of a tobramycin (Tob)‐loaded tFNAs complex (TTob), enveloped in a neutrophil‐platelet hybrid membrane (NPM) derived from neutrophil vesicles (NVs) and platelet vesicles (PVs).^[^ ^22^, ^23^ ^]^ By incorporating the characteristics of both neutrophil and platelet membranes, the nanocarrier manages to acquire an intact lipid membrane and multiple functions that resemble those of the original cells.^[^ ^24^ ^]^ The membrane from NVs enables the nanocarriers to target inflammation following a chemotactic gradient and neutralize toxins,^[^ ^25^ ^]^ while the membrane from PVs enhances their binding capacity to bacteria. In addition, the layer of natural cell membrane provides robust protection against immune response and biofouling process in the physiological system, which further improves the accumulation and motion behavior of TTob@NPM in the infection site. Moreover, the release of TTob could be triggered by toxin‐mediated membrane lysis. In addition, benefitting from the immunomodulation potential of tFNAs, TTob@NPM is capable of coordinating macrophage polarization and MDSCs recruitment to restore the immunosuppressive microenvironment caused by *P. aeruginosa* infection (**Figure** **1**).

By integrating targeted antibiotic delivery, toxin/bacteria clearance, and immunomodulation into a single therapeutic agent, this innovative approach aims to tackle *P. aeruginosa*‐induced VAP.

## 2 Results

### 2.1 Preparation and Characterization of TTob@NPM

**Figure 2:**
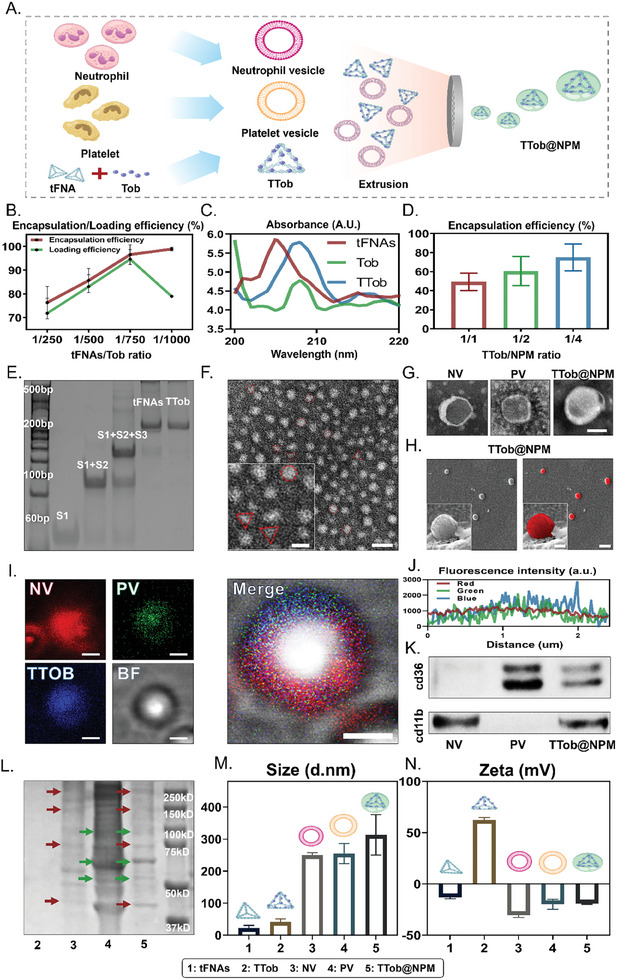
Preparation and characterization of TTob@NPM. A) Schematic display of the fabrication of TTob@NPM. B) The encapsulation and loading efficiency of TTob with different ratios of tFNAs to Tob. C) The absorbance spectra of tFNAs, Tob, and TTob. D) The encapsulation efficiency of TTob@NPM with different ratios of TTob to NPM. E) Confirmation of the successful synthesis of tFNAs and TTob by PAGE. F) The TEM images showed a circle‐like shaped structure of TTob and a triangle‐like shaped structure of tFNAs. Scale bars are 25 nm (left) and 50 nm (right). G) The TEM images of NV, PV, and TTob@NPM. Scale bar is 100 nm. H) SEM images of TTob@NPM. Scale bars are 125 nm (left) and 500 nm (right). I) CLSM images of fusion among DiI‐labeled NV (red), DiO‐labeled PV (green), and Cy5‐labeled TTob (blue) during the preparation of TTob@NPM. Scale bars are 150 nm. BF, bright field. J) The fluorescence intensity spectra of DiI‐labeled NV (red), DiO‐labeled PV (green), and Cy5‐labeled TTob (blue). K) Western blotting analysis of membrane protein cd36 and cd11b in NV, PV, and TTob@NPM. L) SDS‐PAGE analysis of total membrane protein isolated from TTob, NV, PV, and TTob@NPM. The red arrows indicate bands occurring in both NV and TTob@NPM. The green arrows indicate bands occurring in both PV and TTob@NPM. M) The hydrodynamic sizes of tFNAs, TTob, NV, PV, and TTob@NPM. N) The zeta potentials of tFNAs, TTob, NV, PV, and TTob@NPM. The error bars represented the SD. *n* ≥ 3. tFNAs, tetrahedral framework nucleic acids; TTob, tobramycin‐loaded tFNAs complex; NV, neutrophil vesicle; PV, platelet vesicle; TTob@NPM, TTob encapsulated with neutrophil and platelet hybrid membranes.

As shown in **Figure** **2**A, TTob@NPM was constructed by encapsulating TTob into NPM with reduplicative extrusion. First, TTob was designed and assembled for both antibiotic loading and bacteria killing. According to the standard curves concerning the absorbance‐concentration relationship of tFNAs (Figure S1A, Supporting Information) and Tob (Figure S1B, Supporting Information), the encapsulation and loading efficiency at different tFNAs:Tob ratios were calculated. The ratio 1:750 was selected based on overall Tob utilization with an encapsulation efficiency of 96.6 ± 4.2% and loading efficiency of 94.6 ± 4.1% (Figure 2B). TTob showed characteristic absorbance peaks in ultraviolet spectrometry (Figure 2C). Then, NVs and PVs were extruded to form NPM, which was used to envelope TTob particles to construct TTob@NPM for drug delivery. The encapsulation efficiency of NPM exhibited an upward trend with the increase of NPM concentration, and the ratio 1:4 (TTob:NPM) was selected with an encapsulation of 75.0 ± 14.1% (Figure 2D). The successful synthesis of tFNAs and TTob was confirmed with polyacrylamide gel electrophoresis (PAGE) (Figure 2E). Transmission electron microscopy (TEM) was used to further elucidate the morphology of tFNAs (indicated by triangles) and TTob (indicated by circles) (Figure 2F). The TTob@NPM complex was visualized by TEM and showed a spherical core–shell structure (Figure 2G and Figure S1C, Supporting Information). Subsequent analyses using scanning electron microscope (SEM) (Figure 2H) and confocal laser scanning microscope (CLSM) (Figure S1D, Supporting Information) indicated the typical phospholipid bilayer structure and smooth surface of TTob@NPM. To further examine the successful fabrication of TTob@NPM, fluorescence colocalization of DiI‐labeled NV (red), DiO‐labeled PV (green), and Cy5‐labeled TTob (blue) were imaged (Figure 2I), reflecting a notable fusion of the three materials (Figure 2J). Furthermore, immunoblotting (Figure 2K) and SDS‐PAGE (Figure 2L) confirmed the presence of key membrane proteins on TTob@NPM, which proved the translocation of NPM. Dynamic light scattering (DLS) measurements revealed that the hydrodynamic diameter (Figure 2M) and surface zeta potential (Figure 2N) of the TTob@NPM were 313.0 ± 62.8 nm and −19.0 ± 0.8 mV. Hemolysis assay demonstrated that the TTob@NPM maintained a low hemolysis rate (Figure S1E, Supporting Information). Moreover, the TTob@NPM presented satisfactory stability in PBS at 4 °C within 7 d (Figure S1F, Supporting Information), in *P. aeruginosa* supernatant at 37 °C within 2 h (Figure S1G, Supporting Information), and in 10% FBS at 37 °C within 12 h (Figure S1H, Supporting Information).

To sum up, a series of observations demonstrated the successful synthesis as well as multiple physicochemical features of TTob@NPM.

### 2.2 Antibacterial Activity against Planktonic Bacteria and Biofilm In Vitro

**Figure 3:**
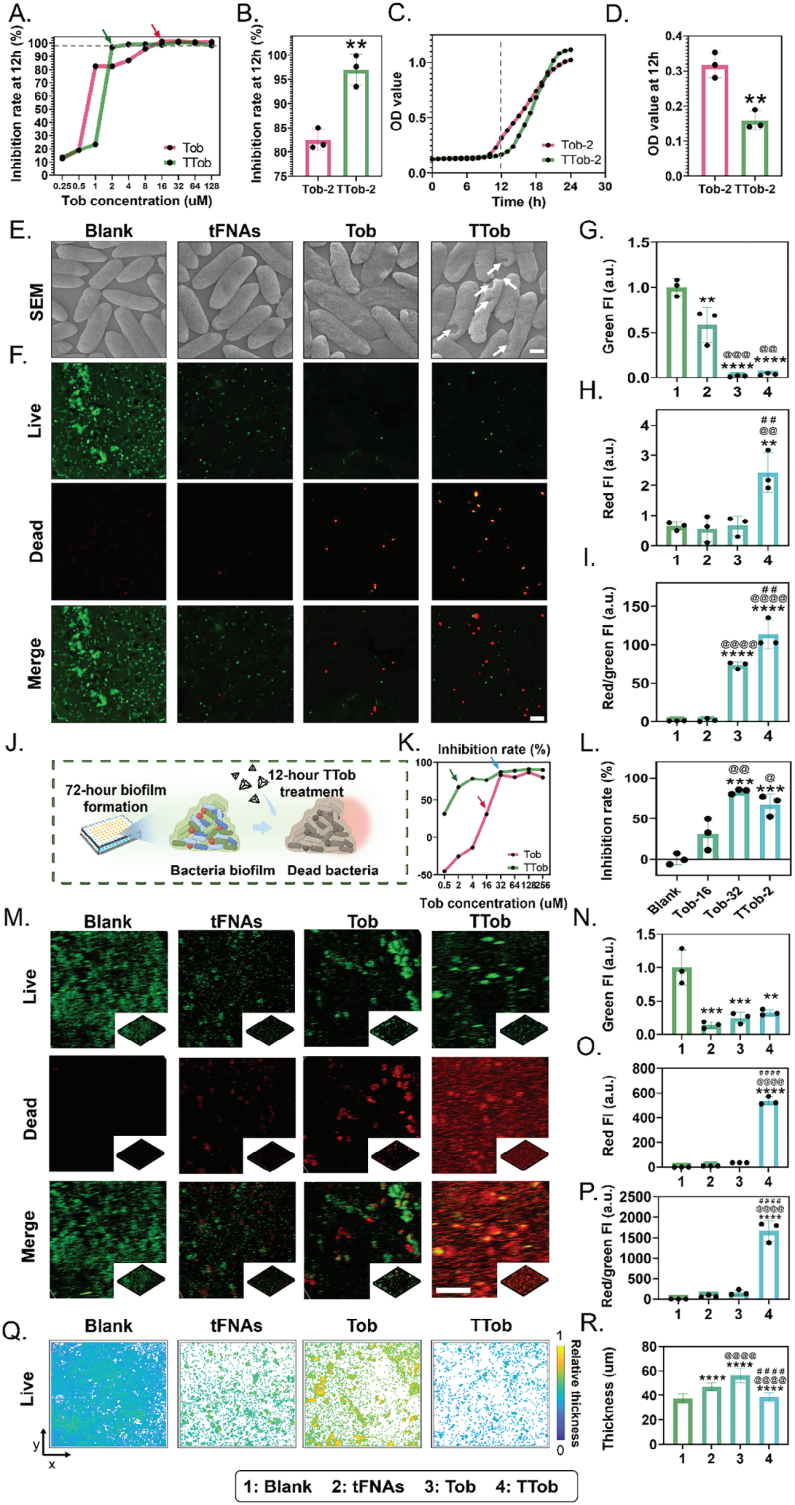
Antimicrobial activity of TTob against planktonic *P. aeruginosa* cultures and *P. aeruginosa* biofilms. A) Inhibition effect on *P. aeruginosa* growth with different Tob and TTob concentration at 12 h. The dashed line indicates 97% inhibition rate. The green and red arrows indicate MIC of TTob and Tob, respectively. B) The inhibition rate was measured after *P. aeruginosa* cultures were treated with 2 × 10^−6^ m Tob (Tob‐2) and 2 × 10^−6^ m TTob (TTob‐2) for 12 h. C) The growth curves of *P. aeruginosa* with Tob‐2 and TTob‐2 treatments across 24 h. The dashed line indicates 12 h. D) The OD600 was measured after *P. aeruginosa* cultures were treated with Tob‐2 and TTob‐2 for 12 h. E) SEM images of *P. aeruginosa* morphology changes after TSB medium (Blank), 100 × 10^−9^ m tFNAs (tFNAs), 2 × 10^−6^ m Tob (Tob), and 2 × 10^−6^ m TTob (TTob) treatments. Scale bar is 500 nm. F) Images of live/dead bacteria staining for *P. aeruginosa* cultures, in which green signals represent the surviving bacteria while red signals indicate dead bacteria. Scale bar is 40 µm. G) Relative green fluorescence intensity of *P. aeruginosa* cultures after different treatments. H) Relative red fluorescence intensity of *P. aeruginosa* cultures after different treatments. I) Relative red/green fluorescence intensity of *P. aeruginosa* cultures after different treatments. J) Schematic illustration of the experiment design. K) Inhibition effect on *P. aeruginosa* biofilms growth with different Tob and TTob concentration at 12 h. The green arrow indicates 2 × 10^−6^ m TTob, the red arrow indicates 16 × 10^−6^ m Tob, and the blue arrow indicates that TTob and Tob have comparable inhibition rate at 32 × 10^−6^ m. L) The inhibition rate was measured after *P. aeruginosa* biofilms were treated with TSB medium (Blank), 16 × 10^−6^ m Tob (Tob‐16), 32 × 10^−6^ m Tob (Tob‐32), and 2 × 10^−6^ m TTob (TTob‐2) for 12 h. M) Images of live/dead bacteria staining for *P. aeruginosa* biofilms after TSB medium (Blank), 100 × 10^−9^ m tFNAs (tFNAs), 2 × 10^−6^ m Tob (Tob), and 2 × 10^−6^ m TTob (TTob) treatments, in which green signals represent the surviving bacteria while red signals indicate dead bacteria. Scale bar is 100 µm. FI, fluorescence intensity. N) Relative green fluorescence intensity of *P. aeruginosa* biofilms after different treatments. O) Relative red fluorescence intensity of *P. aeruginosa* biofilms after different treatments. P) Relative red/green fluorescence intensity of *P. aeruginosa* biofilms after different treatments. Q) Relative density and thickness of *P. aeruginosa* biofilm after different treatments visualized by scatter plots. R) Statistical analysis of biofilm thickness after different treatments. The error bars represented the SD. ^*^ compared to the first group; ^*^ *p* < 0.05, ^**^ *p* < 0.01, ^***^ *p* < 0.001, and ^****^ *p* < 0.0001. ^@^ compared to the second group; ^@^ *p* < 0.05, ^@@^ *p* < 0.01, ^@@@^ *p* < 0.001, and ^@@@@^ *p* < 0.0001. ^#^ compared to the third group; ^#^ *p* < 0.05, ^##^ *p* < 0.01, ^###^ *p* < 0.001, and ^####^ *p* < 0.0001. *n* ≥ 3. Blank, TSB medium; tFNAs, tetrahedral framework nucleic acids; Tob, tobramycin; TTob, tobramycin‐loaded tFNAs complex.

TTob was the key component of TTob@NPM responsible for planktonic bacteria killing and biofilm elimination. To investigate the effectiveness of Tob and TTob in eradicating planktonic bacteria, *P. aeruginosa* was cultured with varying concentrations of Tob and TTob for 12 h, when *P. aeruginosa* growth reached the end of the logarithmic phase (Figure S2A, Supporting Information). The primary objective was to ascertain the minimum inhibitory concentration (MIC) of Tob (MIC_Tob_) necessary to hinder 97% of bacterial growth. Notably, Tob alone exhibited a MIC value of 16 × 10^−6^ m (indicated by the red arrow in **Figure** **3**A). When TTob was introduced, it displayed an enhanced antibacterial activity, as evidenced by a substantial reduction in the MIC value to 2 × 10^−6^ m (indicated by the green arrow in Figure 3A). Thus, 2 × 10^−6^ m (1/8 × MIC_Tob_) was selected for Tob concentration in Tob (Tob‐2) and TTob (TTob‐2) group for subsequent experiments, in which TTob‐2 (Inhibition rate = 97.03 ± 3.23%; OD value = 0.16 ± 0.03) showed higher inhibition rate (Figure 3B and Figure S2B, Supporting Information) and lower OD value (Figure 3C,D) compared with Tob‐2 (Inhibition rate = 82.51 ± 2.18%; OD value = 0.32 ± 0.04) group at 12 h. On the other hand, due to the fast and efficient antibacterial activity of TTob‐2, Tob‐2 presented a more lasting antibacterial activity after 20 h. When the concentration of Tob was equally set in the Tob and TTob group ranging from 4 to 16 × 10^−6^ m, the TTob group had an enhanced or at least comparable antibacterial effect compared to the Tob group (Figure S2C–H, Supporting Information). Planktonic bacteria were cultured with therapeutics for 12 h before being examined by SEM, which demonstrated that the bacteria wall became crumpled but remained intact after Tob treatment, while the TTob treatment led to a partially destroyed and deformed bacteria wall (indicated by arrows). The other treatment did not lead to obvious changes in bacterial morphology (Figure 3E and Figure S2I, Supporting Information). Live/dead staining (Figure 3F), with green and red indicating the surviving bacteria (Figure 3G) and dead bacteria (Figure 3H) respectively, showed that TTob treatment limited bacteria growth and proliferation, leading to a significant increase in dead/live ratio and favorable antibacterial property (Figure 3I).

Furthermore, apart from their planktonic form, *P. aeruginosa* can form biofilms, which are notorious for their antimicrobial resistance. Hence, the antibacterial activity of TTob against biofilms was investigated. *P. aeruginosa* was cultured for 72 h (Figure 3J), allowing the formation of robust biofilms that exhibited significant resistance to Tob. 16 × 10^−6^ m (1 × MIC_Tob_, indicated by red arrow in Figure 3K and Figure S3A, Supporting Information) Tob (OD value = 0.69 ± 0.19; Inhibition rate = 31.34 ± 19.26%) alone demonstrated no obvious antibacterial effect compared to control group (OD value = 1.00 ± 0.09; Inhibition rate = 0.00 ± 6.83%), while 16 × 10^−6^ m TTob (OD value = 0.23 ± 0.05; Inhibition rate = 76.53 ± 5.26%) led to a significant reduction in bacteria biomass, which was 2.4 times greater than that of Tob group. Tob and TTob had a comparable antibacterial effect when Tob concentration reached 32 × 10^−6^ m (indicated by blue arrow in Figure 3K and Figure S3A, Supporting Information). It was noteworthy that even 2 × 10^−6^ m (1/8 × MIC_Tob_, indicated by green arrow in Figure 3K and Figure S3A, Supporting Information) TTob (OD value = 0.33 ± 0.13; Inhibition rate = 67.18 ± 13.05%) achieved favorable antibacterial effect (Figure 3L). Therefore, Tob at the concentration of 2 × 10^−6^ m (1/8 × MIC_Tob_) was set for subsequent experiments. Live/dead staining showed that TTob treatment achieved satisfactory antibacterial property (Figure 3M–P). Besides, the density and thickness of live bacteria biofilm were assessed, which showed that TTob treatment led to limited biofilm formation with more sparse bacteria colonization and thinner biofilm formation (Figure 3Q,R). Previous studies have indicated that inadequate delivery of antimicrobials at sublethal doses can promote biofilm growth.^[^ ^26^, ^27^ ^]^ This phenomenon helps to explain the observed proliferation of bacterial biofilms at low Tob concentrations and underscores the importance of effective antibiotic delivery through TTob. When the concentration of Tob was equally set in Tob and TTob group ranging from 0.5 to 256 × 10^−6^ m, TTob group had an enhanced or at least comparable antibacterial effect compared to Tob group (Figure S4A–H, Supporting Information).

Collectively, as the core component of TTob@NPM, TTob exhibited improved antibacterial activity against both planktonic bacteria and biofilm, making it a promising candidate for treating bacterial infections.

### 2.3 Targeted Antibiotic Delivery and Toxin/Bacteria Clearance of TTob@NPM

**Figure 4:**
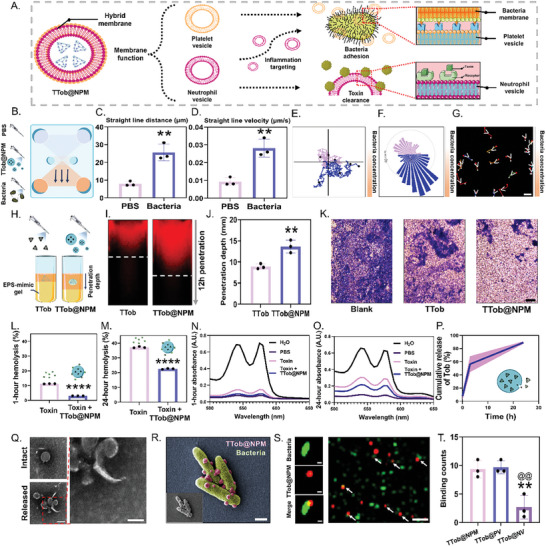
Migration, toxin/bacteria clearance and drug release behavior of TTob@NPM in vitro. A) Schematic illustration of hybrid membrane function of TTob@NPM. B) Schematic illustration of the chemotaxis assay. C) Straight line distance, defined as the distance between the last spot of the track and the first spot of the track in time, of TTob@NPM toward PBS and bacteria. D) Straight line velocity, the straight line distance divided by the time difference, of TTob@NPM toward PBS and bacteria. E) Migration of TTob@NPM in a chemotaxis chamber with *P. aeruginosa* on the lower side. Blue and purple lines show the migration tracks of TTob@NPM toward *P. aeruginosa* (down) and PBS (up), respectively. F) Migration counts of TTob@NPM in a chemotaxis chamber with *P. aeruginosa* on the lower side. Blue and purple bars show the migration count of TTob@NPM toward *P. aeruginosa* (down) and PBS (up), respectively. G) Representative optical trajectories of TTob@NPM migration that were longer than 150 µm with *P. aeruginosa* on the lower side. Red and white dots show the start points and end points of the trajectories, respectively. White arrows indicate moving direction. Scale bar is 10 µm. H) Schematic illustration of the EPS‐mimic gel penetration assay. I) Fluorescence imaging of EPS‐mimic gel after TTob@NPM and TTob treatments. The dashed lines indicate the most front penetration margin. J) Statistical analysis of penetration depth after TTob and TTob@NPM treatments. K) Representative crystal violet stained‐biofilm images after different treatments. Scale bar is 100 µm. L) 1‐h and M) 24‐h hemolysis percentages after different treatments. N) 1‐h and O) 24‐h absorbance spectra of oxyhemoglobin after different treatments. PBS without toxin was used as a negative control. The absorbance spectra of oxyhemoglobin after sonicating the 5% RBC solution for 5 min in H_2_O was used as a positive control. P) In vitro release kinetics of Tob from TTob@NPM in *P. aeruginosa* supernatant. Q) TEM images of intact and released form of TTob@NPM. Scale bars are 25 nm (left) and 100 nm (right). R) SEM images of bacteria adhesion. Green indicates *P. aeruginosa* and red indicates TTob@NPM. Scale bar is 500 nm. S) Fluorescent imaging of bacteria adhesion. Green fluorescence indicates *P. aeruginosa* and red fluorescence indicates TTob@NPM. Scale bars are 1 µm (left) and 200 µm (right). T) Statistical analysis of bacteria adhesion after different treatments. The error bars represented the SD. ^*^ compared to the first group; ^*^ *p* < 0.05, ^**^ *p* < 0.01, ^***^ *p* < 0.001, and ^****^ *p* < 0.0001. ^@^ compared to the second group; ^@^ *p* < 0.05, ^@@^ *p* < 0.01, ^@@@^ *p* < 0.001, and ^@@@@^ *p* < 0.0001. *n* ≥ 3. TTob, tobramycin‐loaded tFNAs complex; TTob@NV, TTob encapsulated with neutrophil vesicle; TTob@PV, TTob encapsulated with platelet vesicle; TTob@NPM, TTob encapsulated with neutrophil and platelet hybrid membranes.

Once TTob was enveloped within NPM to form TTob@NPM, various functions were acquired to further improve the antibacterial activity. The underlying hypothesis suggests that platelet membrane improves the ability of nanocarriers to bind to bacteria, while the neutrophil membrane allows them to specifically target inflammation and neutralize toxins. In addition, TTob release can be initiated through toxin‐induced membrane lysis (**Figure** **4**A).

First, the chemotactic function of TTob@NPM was evaluated using µ‐slide chemotaxis assay (Figure 4B). The straight line distance (the distance between the last spot of the track and the first spot of the track in time, Figure 4C) and straight line velocity (the straight line distance divided by the time, Figure 4D) of TTob@NPM toward higher gradient of *P. aeruginosa* (25.670 ± 4.750 µm and 0.028 ± 0.005 µm s^−1^) were significantly increased than that toward lower gradient (7.970 ± 1.340 µm and 0.009 ± 0.002 µm s^−1^). Notably, the accumulated distance (the full distance the particle traveled throughout the track, Figure S5A, Supporting Information) and velocity (the accumulated distance divided by the time, Figure S5B, Supporting Information) between two groups had no significant difference, while the directionality (the straight line distance divided by accumulated distance, Figure S5C, Supporting Information) of TTob@NPM toward higher bacterial gradient (0.167 ± 0.032) was significantly increased than that toward lower gradient (0.051 ± 0.010), which illustrated that unlike the nanorobot with self‐propulsion design harvesting thrust from either localized chemical reactions or from external stimuli to accelerate individual motion, the cell membrane coating improved the overall motion of TTob@NPM toward the attractant gradient rather than the motility of an individual specimen. The efficient migration of TTob@NPM toward higher gradient of *P. aeruginosa* was visualized in migration tracks (Figure 4E), migration counts (Figure 4F), and representative optical trajectories (Figure 4G and Video S1, Supporting Information). *P. aeruginosa* biofilms can limit antibiotic penetration and reduce antibacterial efficacy due to EPS. Thus, the penetration of TTob@NPM was investigated in an EPS‐mimic gel (Figure 4H). The penetration depth of TTob@NPM was 13.650 ± 1.500 mm, which was ≈1.5 times greater than that of TTob (8.900 ± 0.620 mm) (Figure 4I,J). Positively charged TTob could interact with negatively charged components of the biofilm matrix, such as polysaccharides and extracellular DNA, which reduced the diffusion through the biofilm.^[^ ^28^ ^]^ Compared with TTob, the enhanced penetration of TTob@NPM was probably attributed to its negative charge, which was less affected by negatively charged components of the matrix. Besides, notable biofilm elimination was detected in the TTob@NPM group (Figure 4K), indicating that TTob@NPM can enhance antibiotic delivery into biofilms.

After validation of targeted antibiotic delivery, we proceeded to evaluate the detoxification capabilities of TTob@NPM. In our experimental setup, leucocidin was used as a representative PFT to assess the detoxification potential of TTob@NPM. TTob@NPM was subjected to treatment with leucocidin and subsequently incubated with a 5% solution of pure red blood cells (RBCs) for durations of 1 and 24 h, respectively. Following the incubation period, the RBC solution underwent centrifugation, and the absorbance of the resulting supernatant was measured at 540 nm. This measurement served as an estimation of the extent of hemolysis occurring in the system. Substantial lower hemolysis was observed in Toxin + TTob@NPM group after 1 h (Figure 4L) and 24 h (Figure 4M) incubation, which was further confirmed by the absorbance spectra of oxyhemoglobin (Figure 4N,O, respectively). Next, the drug release profile was evaluated. As shown in Figure 4P, Tob could be gradually released from TTob@NPM after incubated with *P. aeruginosa* supernatant. TEM images showed the disintegration of TTob@NPM and the release of TTob (Figure 4Q).

Afterward, TTob@NPM was assessed for selective binding to *P. aeruginosa*. TTob@NPM was incubated in the *P. aeruginosa* suspension followed by SEM imaging, which proved the pathogen binding capacity of TTob@NPM (Figure 4R). Microscopic fluorescence imaging further illustrated the specific binding between *P. aeruginosa* (green) and TTob@NPM (red) (Figure 4S). The binding counts in TTob@NV group were significantly lower than that in TTob@NPM and TTob@PV group (Figure 4T), indicating the central role of PV in bacteria binding.

In sum, these results demonstrated the targeted antibiotic delivery and concurrent toxin/bacteria removal capacity of TTob@NPM.

### 2.4 Lung Distribution and Retention of TTob@NPM In Vivo

**Figure 5:**
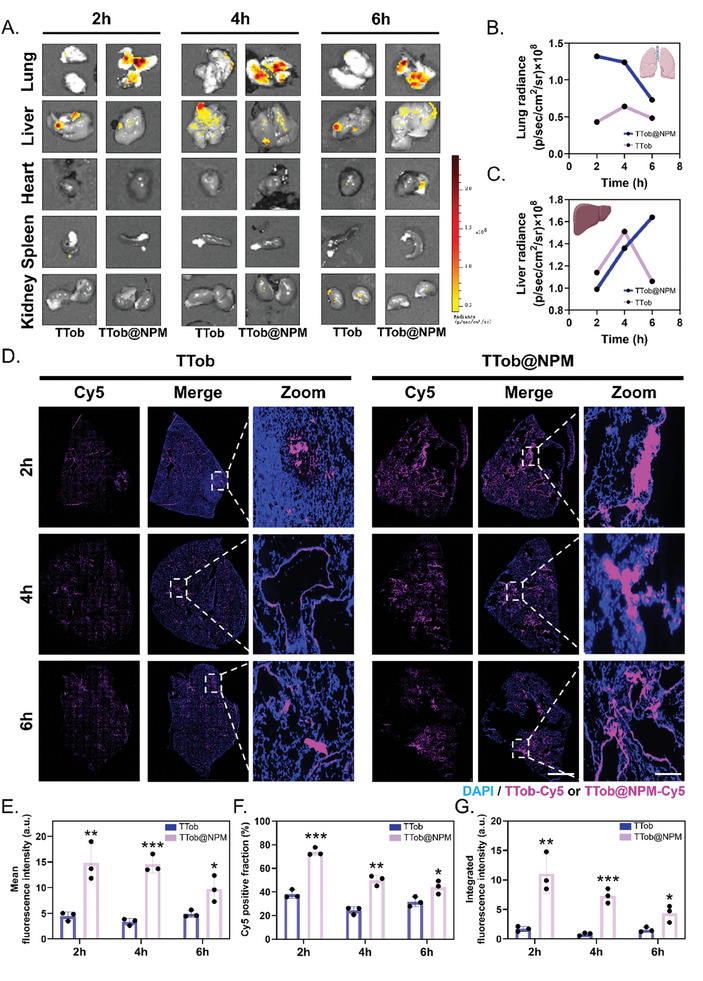
Lung distribution and retention of TTob@NPM in vivo. A) Ex vivo fluorescent imaging of main organs at various timepoints after intranasal administration of TTob and TTob@NPM. B) Quantitative analysis for ex vivo fluorescent imaging of lungs at various timepoints. C) Quantitative analysis for ex vivo fluorescent imaging of livers at various timepoints. D) Lung distribution of TTob and TTob@NPM. Purple fluorescence (Cy5) indicates TTob or TTob@NPM. Blue fluorescence (DAPI) indicates nuclei. Scale bars are 450 µm (middle) and 2 mm (right). E) Statistical analysis of TTob@NPM and TTob's mean fluorescence intensity in lungs. F) Statistical analysis of Cy5 positive fraction in lungs. G) Statistical analysis of TTob@NPM and TTob's integrated fluorescence intensity in lungs. The error bars represented the SD. ^*^ compared to the first group; ^*^ *p* < 0.05, ^**^ *p* < 0.01, ^***^ *p* < 0.001, and ^****^ *p* < 0.0001. *n* ≥ 3. TTob, tobramycin‐loaded tFNAs complex; TTob@NPM, TTob encapsulated with neutrophil and platelet hybrid membranes.

The lung distribution of TTob@NPM was examined after intranasal administration to *P. aeruginosa*‐infected mice lungs. Fluorescence imaging of the harvested livers showed that TTob were rapidly metabolized in the liver with fluorescence intensity reached peak at 4 h and decreased dramatically afterward, while fluorescence from TTob@NPM mainly distributed in lungs throughout 6 h and demonstrated a continuous upward trend within 6 h in livers (**Figure** **5**A–C). Further analysis of lung distribution was visualized by immunofluorescence of the lungs. The intense fluorescence of TTob@NPM retained for at least 6 h and permeated among cells. In contrast, the fluorescence of TTob was barely detected even at 2 h (Figure 5D and Figure S5D, Supporting Information), which was further proved by statistical analysis of mean fluorescence intensity (Figure 5E), Cy5 positive fraction (Figure 5F) and integrated fluorescence intensity (Figure 5G), revealing the superior properties of TTob@NPM in prolonged lung retention time and greater permeability to deeper infection sites.

### 2.5 Safety Assessment of TTob@NPM In Vivo

To assess the biosafety of TTob@NPM, additional experiments were conducted to observe if there was any toxic effect after intranasal administration. The serum biochemical parameters, routine blood examination indicators, and bronchoalveolar lavage fluid (BALF) protein levels of mice administered with TTob@NPM showed no significant difference compared to those of mice in blank group (Figure S6A–H, Supporting Information). Meanwhile, the changes of cytokine levels in blood and BALF were not statistically significant (Figure S6I, Supporting Information). The images of hematoxylin and eosin (H&E)‐stained slices revealed no visible injury or inflammation in major organs (Figure S6J, Supporting Information).

### 2.6 Therapeutic and Immunomodulatory Effects of TTob@NPM on *P. aeruginosa*‐Induced Acute Lung Infection In Vivo

**Figure 6:**
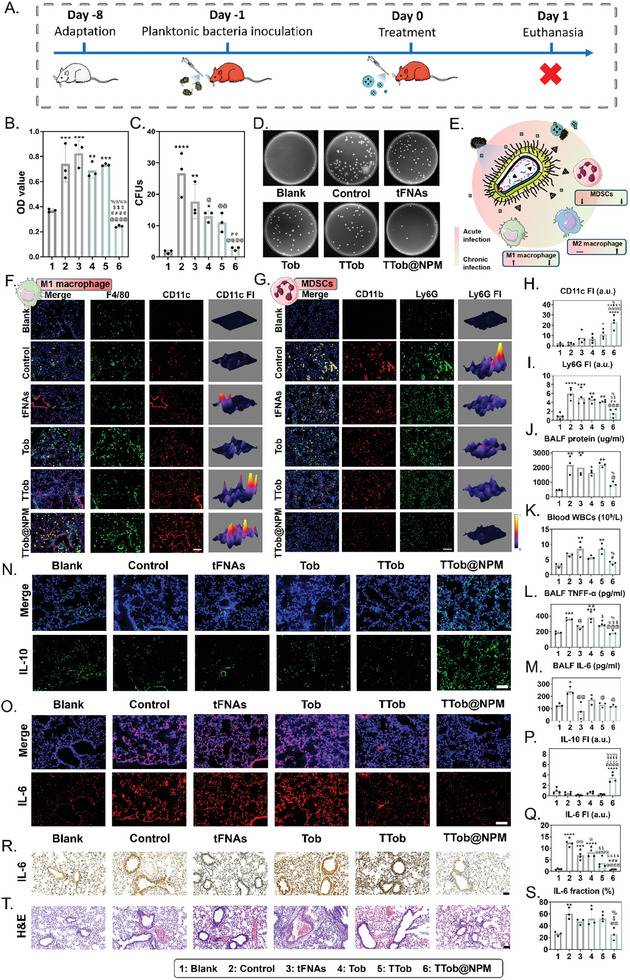
Therapeutic and immunomodulatory effects of TTob@NPM on *P. aeruginosa*‐induced acute lung infection in vivo. A) Schematic illustration of animal model establishment and drug administration to acute *P. aeruginosa*‐infected mouse. B) The OD600 of 12‐h cultured acute infected‐lung homogenization after different treatments. C) CFUs of viable bacteria in acute infected‐lung homogenization after different treatments. D) Results of the spread plate assay for acute infected‐lung homogenization after different treatments. E) Schematic illustration of TTob@NPM's immunomodulatory effect on *P. aeruginosa*‐induced lung infection. F) Immunofluorescence staining of F4/80 (green), CD11c (red), and DAPI (blue) expression. Co‐localization of the three signals indicate M1‐like macrophages. FI, fluorescence intensity. Scale bar is 100 µm. G) Immunofluorescence staining of CD11b (red), Ly6G (green), and DAPI (blue) expression. Co‐localization of the three signals indicates MDSCs. FI, fluorescence intensity. Scale bar is 100 µm. H) Quantitative analysis for immunofluorescence staining of M1‐like macrophages level. I) Quantitative analysis for immunofluorescence staining of MDSCs level. J) BALF protein levels after different treatments. K) Blood WBC counts after different treatments. L) BALF TNF‐α levels after different treatments. M) BALF IL‐6 levels after different treatments. N) Immunofluorescence staining of IL‐10 (green) and DAPI (blue) expression. FI, fluorescence intensity. Scale bar is 100 µm. O) Immunofluorescence staining of IL‐6 (red) and DAPI (blue) expression. FI, fluorescence intensity. Scale bar is 100 µm. P) Quantitative analysis for immunofluorescence staining of IL‐10 level. Q) Quantitative analysis for immunofluorescence staining of IL‐6 level. R) Representative images for immunohistochemical analysis of lung IL‐6 after different treatments. Scale bar is 100 µm. S) Quantitative analysis for lung immunohistochemical IL‐6 level after different treatments. T) Representative images for H&E staining of lungs after different treatments. Scale bar is 100 µm. The error bars represented the SD. ^*^ compared to the first group; ^*^ *p* < 0.05, ^**^ *p* < 0.01, ^***^ *p* < 0.001, and ^****^ *p* < 0.0001. ^@^ compared to the second group; ^@^ *p* < 0.05, ^@@^ *p* < 0.01, ^@@@^ *p* < 0.001, and ^@@@@^ *p* < 0.0001. ^#^ compared to the third group; ^#^ *p* < 0.05, ^##^ *p* < 0.01, ^###^ *p* < 0.001, and ^####^ *p* < 0.0001. ^$^ compared to the fourth group; ^$^ *p* < 0.05, ^$$^ *p* < 0.01, ^$$$^ *p* < 0.001, and ^$$$$^ *p* < 0.0001. ^%^ compared to the fifth group; ^%^ *p* < 0.05, ^%%^ *p* < 0.01, ^%%%^ *p* < 0.001, and ^%%%%^ *p* < 0.0001. *n* ≥ 3. Blank, healthy group; Control, infected group; tFNAs, tetrahedral framework nucleic acids; Tob, tobramycin; TTob, tobramycin‐loaded tFNAs complex; TTob@NPM, TTob encapsulated with neutrophil and platelet hybrid membranes.

The progression of *P. aeruginosa* infection in the lungs follows a gradual course, with the acute phase characterized by the presence of individual bacteria, while the chronic phase occurs once biofilm formation takes place. Here, TTob@NPM was employed in an acute lung infection model in mice. To establish the acute lung infection model, mice were inoculated with planktonic bacteria following the procedure outlined in **Figure** **6**A. The bacterial burden in the lungs was quantified by assessing the OD value of cultured bacterial suspension and colony‐forming units (CFUs) obtained from lung homogenization. Following treatment, the TTob@NPM group exhibited the lowest bacterial burden, as evidenced by a threefold reduction in both the OD value of 12‐h cultured bacterial suspension (Figure 6B) and the bacterial growth on agar plates (Figure 6C,D) compared to the control group.

In *P. aeruginosa* infection, the virulence factors produced by the bacteria induce an abnormal host immune response, which hampers the function of macrophages responsible for rapid bacterial clearance. Considering the immunomodulatory potential of tFNAs, the impact of TTob@NPM on macrophage polarization and the immune microenvironment was further investigated in vivo (Figure 6E). As depicted in Figure 6F and Figure S7A (Supporting Information), M1‐like macrophages (labeled as F4/80+/CD11c+) and M2‐like macrophages (labeled as F4/80+/CD206+) were identified. The results demonstrated that TTob@NPM treatment led to an upregulation of M1‐like macrophages, which were distributed throughout the infection sites (Figure 6H). Meanwhile, few M2‐like macrophages were detected in all groups (Figure S7B, Supporting Information). Notably, the immunosuppressive cell MDSCs were significantly less enriched in TTob@NPM group compared to control group (Figure 6G,I). These findings revealed the immunomodulatory function of TTob@NPM in promoting M1‐like macrophages to clear bacteria and reverse the immunosuppressive microenvironment. Next, several physiological markers that represent infection were assessed. Statistically significant decreases were observed for BALF protein levels (Figure 6J) and blood white blood cell (WBC) count (Figure 6K) in TTob@NPM group. Furthermore, a decrease in the levels of pro‐inflammatory cytokines TNF‐α (Figure 6L) and IL‐6 (Figure 6M) was observed in the TTob@NPM group. Immunofluorescence images showing upregulated anti‐inflammatory cytokine IL‐10 (Figure 6N,P) and downregulated IL‐6 (Figure 6O,Q) and in the TTob@NPM group. These findings were further supported by immunohistochemical staining of IL‐6 (Figure 6R) and semi‐quantitative analysis (Figure 6S) indicated reduced expression of IL‐6 in the TTob@NPM group. These results suggest that TTob@NPM treatment efficiently alleviated inflammation at the early stage of infection. H&E‐stained images of lung sections validated the reduction in inflammatory cell infiltration and hemorrhage in the TTob@NPM group (Figure 6T). This provides additional evidence for the effectiveness of TTob@NPM in mitigating the inflammatory response associated with the infection.

### 2.7 Therapeutic and Immunomodulatory Effects of TTob@NPM on *P. aeruginosa*‐Induced Chronic Lung Infection In Vivo

**Figure 7:**
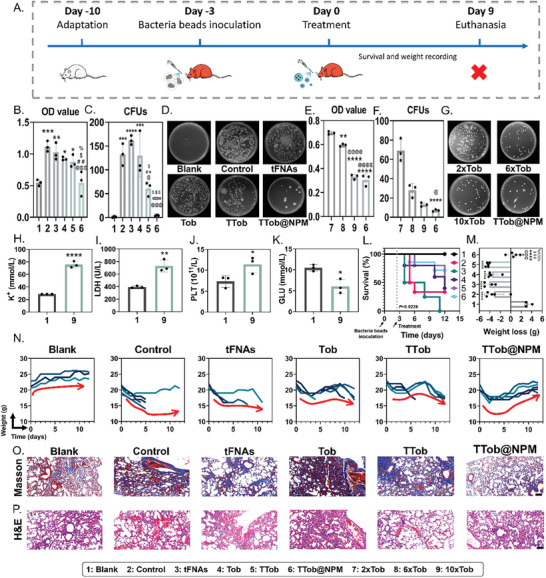
Therapeutic effect of TTob@NPM on *P. aeruginosa*‐induced acute lung infection in vivo. A) Schematic illustration of animal model establishment and drug administration to chronic *P. aeruginosa*‐infected mouse. B) The OD600 of 12‐h cultured chronic infected‐lung homogenization after different treatments. C) CFUs of viable bacteria in chronic infected‐lung homogenization after different treatments. D) Representative images of the spread plate assay for chronic infected‐lung homogenization after different treatments. E) The OD600 of 12‐h cultured chronic infected‐lung homogenization after high‐dose Tob, and TTob@NPM treatments. F) CFUs of viable bacteria in chronic infected‐lung homogenization after high‐dose Tob, and TTob@NPM treatments. G) Representative images of the spread plate assay for chronic infected‐lung homogenization after high‐dose Tob, and TTob@NPM treatments. H) Blood K^+^, I) lactate dehydrogenase (LDH), J) platelet (PLT), and K) glucose (GLU) levels after high‐dose Tob administration. L) Kaplan–Meier curve of mouse survival over 12 d with different treatments. The dashed line indicates the start of treatment (day 3). M) The weight changes of mouse over 12 d with different treatments. N) Weight curves of chronic infected mouse over 12 d with different treatments. Red arrows indicate the general trends of weight changes. O) Representative images for Masson's trichrome staining of lungs after different treatments. Scale bar is 100 µm. P) Representative images for H&E staining of lungs after different treatments. Scale bar is 100 µm. The error bars represented the SD. ^*^ compared to the first group; ^*^ *p* < 0.05, ^**^ *p* < 0.01, ^***^ *p* < 0.001, and ^****^ *p* < 0.0001. ^@^ compared to the second group; ^@^ *p* < 0.05, ^@@^ *p* < 0.01, ^@@@^ *p* < 0.001, and ^@@@@^ *p* < 0.0001. ^#^ compared to the third group; ^#^ *p* < 0.05, ^##^ *p* < 0.01, ^###^ *p* < 0.001, and ^####^ *p* < 0.0001. ^$^ compared to the fourth group; ^$^ *p* < 0.05, ^$$^ *p* < 0.01, ^$$$^ *p* < 0.001, and ^$$$$^ *p* < 0.0001. ^%^ compared to the fifth group; ^%^ *p* < 0.05, ^%%^ *p* < 0.01, ^%%%^ *p* < 0.001, and ^%%%%^ *p* < 0.0001. *n* ≥ 3. Blank, healthy group; Control, infected group; tFNAs, tetrahedral framework nucleic acids; Tob, tobramycin; TTob, tobramycin‐loaded tFNAs complex; TTob@NPM, TTob encapsulated with neutrophil and platelet hybrid membranes.

Afterward, a chronic lung infection model was established via bacteria beads (Figure S8A,B, Supporting Information) inoculation according to the procedure shown in **Figure** **7**A. TTob@NPM treatment led to the lowest bacterial burden in both 12‐h cultured bacteria suspension (Figure 7B) and plates (Figure 7C,D), which were twice and 33 times less than that of control group, respectively. Subsequent analyses demonstrated that TTob@NPM treatment could reduce the dosage of antibiotic to 1/10 compared to the control group, yet with desirable therapeutic effect (Figure 7E–G) to avoid toxicity of high‐dose antibiotic administration with higher K^+^ (Figure 7H), lactate dehydrogenase (LDH) (Figure 7I), platelet (PLT) (Figure 7J) levels, and lower glucose (GLU) (Figure 7K) level. Because of the improved therapeutic efficacy of TTob@NPM in biofilm elimination and inflammation control, the mice in TTob@NPM treatment group exhibited a survival rate of 71% under the lethal challenge, which was twice as much as that of the control group (Figure 7L) and showed a gradual increase in their body weight (Figure 7M,N). The effectiveness of the in vivo treatment was assessed further using histological analysis. Images of lung sections stained with Masson's trichrome revealed that, in the TTob@NPM therapy group, the alveolar structure remained unaltered with only mild fibrosis, whereas the other groups displayed significant pulmonary fibrosis (Figure 7O and Figure S9A, Supporting Information). H&E‐stained images of lung sections validated the marked decrease in inflammatory cell infiltration and hemorrhage in TTob@NPM treatment group (Figure 7P).

**Figure 8:**
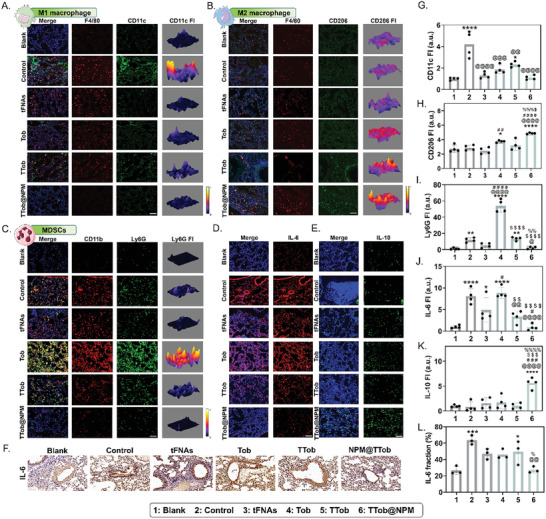
Immunomodulatory effect of TTob@NPM on *P. aeruginosa*‐induced chronic lung infection in vivo. A) Immunofluorescence staining of F4/80 (green), CD11c (red), and DAPI (blue) expression. Co‐localization of the three signals indicate M1‐like macrophages. FI, fluorescence intensity. Scale bar is 100 µm. B) Immunofluorescence staining of F4/80 (red), CD206 (green), and DAPI (blue) expression. Co‐localization of the three signals indicate M2‐like macrophages. FI, fluorescence intensity. Scale bar is 100 µm. C) Immunofluorescence staining of CD11b (red), Ly6G (green), and DAPI (blue) expression. Co‐localization of the three signals indicate MDSCs. FI, fluorescence intensity. Scale bar is 100 µm. D) Immunofluorescence staining of IL‐6 (red) and DAPI (blue) expression. FI, fluorescence intensity. Scale bar is 100 µm. E) Immunofluorescence staining of IL‐10 (green) and DAPI (blue) expression. FI, fluorescence intensity. Scale bar is 100 µm. F) Representative images for immunohistochemical analysis of lung IL‐6 after different treatments. Scale bar is 100 µm. G) Quantitative analysis for immunofluorescence staining of M1‐like macrophages level. FI, fluorescence intensity. H) Quantitative analysis for immunofluorescence staining of M2‐like macrophages level. FI, fluorescence intensity. I) Quantitative analysis for immunofluorescence staining of MDSCs level. FI, fluorescence intensity. J) Quantitative analysis for immunofluorescence staining of IL‐6 level. FI, fluorescence intensity. K) Quantitative analysis for immunofluorescence staining of IL‐10 level. FI, fluorescence intensity. L) Quantitative analysis for lung immunohistochemical IL‐6 level. The error bars represented the SD. ^*^ compared to the first group; ^*^ *p* < 0.05, ^**^ *p* < 0.01, ^***^ *p* < 0.001, and ^****^ *p* < 0.0001. ^@^ compared to the second group; ^@^ *p* < 0.05, ^@@^ *p* < 0.01, ^@@@^ *p* < 0.001, and ^@@@@^ *p* < 0.0001. ^#^ compared to the third group; ^#^ *p* < 0.05, ^##^ *p* < 0.01, ^###^ *p* < 0.001, and ^####^ *p* < 0.0001. ^$^ compared to the fourth group; ^$^ *p* < 0.05, ^$$^ *p* < 0.01, ^$$$^ *p* < 0.001, and ^$$$$^ *p* < 0.0001. ^%^ compared to the fifth group; ^%^ *p* < 0.05, ^%%^ *p* < 0.01, ^%%%^ *p* < 0.001, and ^%%%%^ *p* < 0.0001. *n* ≥ 3. Blank, healthy group; Control, infected group; tFNAs, tetrahedral framework nucleic acids; Tob, tobramycin; TTob, tobramycin‐loaded tFNAs complex; TTob@NPM, TTob encapsulated with neutrophil and platelet hybrid membranes.

From the perspective of immune microenvironment, M1‐like macrophages were barely detected (**Figure** **8**A,G) while M2‐like macrophages aggregated at infection sites after TTob@NPM treatment (Figure 8B,H), which indicated that the inflammation was under control while tissue repair was initiated. Besides, MDSCs were significantly more prominent in Tob group compared to TTob@NPM group (Figure 8C,I), illustrating that TTob@NPM treatment could achieve desirable antibacterial effect without antibiotic‐induced immunosuppression. In addition, a significant decrease in the level of pro‐inflammatory cytokines IL‐6 (Figure 8D,J) with a significant increase in the level of anti‐inflammatory cytokine IL‐10 (Figure 8E,K) were detected in TTob@NPM treatment group. Immunohistochemical staining images of IL‐6 (Figure 8F) and the corresponding semi‐quantitative analysis of stain‐positive areas (Figure 8L) as well revealed that less IL‐6 expression was observed in TTob@NPM treatment group.

## 3 Discussion

VAP caused by *P. aeruginosa* infection has posed a persistent medical challenge, primarily due to the formation of biofilms. Despite the administration of high doses of antibiotics in biofilm‐associated infections, the eradication of bacteria within the biofilms is frequently unsuccessful. This failure can be attributed to the presence of EPS, which acts as a barrier, isolating the bacteria from external antibacterial agents. In addition, the immunosuppressive response of the host further complicates the treatment of VAP induced by *P. aeruginosa* infection.^[^ ^29^ ^]^ Moreover, the administration of high doses of antibiotics often results in severe toxicity to cells and organisms. On the other hand, inadequate antimicrobial administration at sublethal dosage may reversely promote bacteria biofilm growth,^[^ ^26^, ^27^ ^]^ which makes it intractable for clinical practitioners to deal with.

To address the aforementioned challenges associated with traditional antibiotic therapy for VAP, cell membrane‐based nanotherapeutics have emerged as a promising solution with two inherent advantages of cell membranes.^[^ ^30^, ^31^ ^]^ First, by harnessing the biological reactions facilitated by cell membranes, the nanocarriers in our study could be designed to minimize nonspecific uptake and enhance specific targeting capability. This ensured precise delivery of antibiotics direct to the infected lung tissue, thereby circumventing the need for high‐dosage antibiotics that might lead to systemic toxicity. Second, the membrane used for coating nanoparticles in our study could also neutralize toxins and initiate further cellular reaction known as toxin‐mediated membrane lysis. When such process took place, antibiotic‐loaded tFNAs would be released to the targeted sites for bacteria killing and inflammation controlling. By combining the advantages of cell membrane from neutrophils and platelets with the antibacterial efficiency of Tob‐tFNAs complex, TTob@NPM integrates multiple strategies into a single therapeutic agent for VAP treatment.

In particular, we present a bioinspired nanocarrier capable of targeted antibiotic delivery and toxin/bacteria clearance, based on Tob‐loaded tFNAs enveloped with a hybrid membrane derived from neutrophils and platelets. In mouse models of *P. aeruginosa*‐induced lung infection, these nanocarriers could effectively reduce bacterial burden, reverse immunosuppressive microenvironment, and significantly lower animal mortality with negligible toxicity. In ICU settings, chronic colonization by *P. aeruginosa* is believed to impose a profound impact on the morbidity and mortality of VAP patients, leading to prolonged inhaled Tob treatment to prevent exacerbations caused by *P. aeruginosa*.^[^ ^32^, ^33^ ^]^ By leveraging the inflammation‐targeting capability and prolonged tissue retention conferred by the hybrid membrane, these nanocarriers could deliver and release antibiotics to infection sites. Furthermore, the toxin neutralizing and bacteria binding properties of TTob@NPM could enhance its antibacterial capability, resulting in a 10‐time reduction in the antibiotic dosage compared to the control group while maintaining an equivalent therapeutic effect. This approach circumvents the problem of toxicity associated with high‐dose antibiotic administration. In addition, the combination of Tob and tFNAs not only achieves a satisfactory antibacterial effect but also modulates the host immune response. In acute *P. aeruginosa* lung infection, M1‐like macrophages were first activated to facilitate pathogen clearance; as inflammation subsided, these macrophages were polarized to M2‐like macrophages to promote tissue repair. It is worth noting that the recruitment of MDSCs is significantly prohibited throughout the course of *P. aeruginosa* infection, demonstrating that TTob@NPM can achieve a desirable antibacterial effect without inducing immunosuppression at the infection sites.

Although this study is primarily focused on Tob treatment for *P. aeruginosa*, given the fact that one of the most prevalent lung bacterial infections is *P. aeruginosa*‐induced VAP and Tob is usually used for *P. aeruginosa* infections,^[^ ^12^, ^34^ ^]^ the developed nanocarrier system exhibits high versatility and could potentially be extended to other charged antibiotics, enabling the targeting of not only *P. aeruginosa* but also other bacterial species commonly found in mixed infections. In the experiments conducted, the nanocarriers were administered via intranasal instillation of a TTob@NPM suspension. However, it is conceivable to adapt this delivery method for use with a nebulizer. The particle size, stability profiles, and lyophilization potential of the nanocarriers make them suitable for delivery via powder inhalation. This adaptation would enhance the translational potential of TTob@NPM and facilitate its incorporation into an inhaler device, thereby offering a practical and convenient administration route for patients.

The complex characteristics and diversity of the patient‐specific inflammatory network lead to notable differences in patients' response to treatment. Hence, it is crucial to consider customized nanotherapeutic approaches. In contrast to conventional therapies, it is essential to establish a comprehensive treatment system that incorporates both temporal and spatial elements.^[^ ^35^, ^36^ ^]^ TTob@NPM, as an attempt to fulfill the above system, encompasses specific targeting of inflammatory microenvironment as well as controlled release of antibiotic. As emphasized in this study, the targeted delivery of antibiotic to infected sites represents a more effective alternative with less toxicity when compared to conventional approaches. However, it is crucial to assess the potential off‐target effects of the system which may result in unwanted antibiotic accumulation and drug resistance. Hence, real‐time monitoring of these dynamic interactions is imperative to fine‐tune the dosage of nanotherapeutics and optimize therapeutic efficacy at various stages of the disease.^[^ ^37^ ^]^ Inspired from the concept of integrating diagnostic and therapeutic components within a smart nanocarrier, future research could focus on the adjustment of drug dosage by TTob@NPM based on various feedbacks from the microenvironment.

In summary, TTob@NPM has been proved in our study to be effective in treating *P. aeruginosa* induced VAP, and is therefore believed to be a potent antibiotic delivery system with both simplicity and efficacy.

## 4 Experimental Section

### Synthesis of tFNAs and Antibiotic Loading

TM buffer (pH 8.0) was prepared by combining a solution of 10 × 10^−3^ m Tris‐HCl and 50 × 10^−3^ m MgCl_2_. Following that, four distinct single DNA strands (Sangon, China) were added to the TM buffer at a concentration of 1 × 10^−6^ m each to generate tFNAs. The tFNAs were formed by subjecting the mixture to a temperature of 95 °C for 10 min, followed by cooling at 4 °C for 20 min. For more detailed information on the synthesis process, please refer to the previously published research reports.^[^ ^38^, ^39^, ^40^, ^41^ ^]^ The sequences for each DNA strand can be found in Table S1 (Supporting Information). Subsequently, the tFNAs were subjected to vibration with Tob at various ratios (1:250, 1:500, 1:750, and 1:1000) at room temperature for 5 h. The resulting mixture, referred to as TTob, was then incubated at 4 °C for 12 h. To separate the unincorporated Tob molecules, ultrafiltration using a 30 kDa filter was performed. The successful formation of tFNAs and TTob was confirmed through PAGE analysis and absorbance peak scanning. Based on the loaded Tob content, the loading and encapsulation efficiency of TTob at different incubation ratio were determined by an ultra‐microspectrophotometer according to the following formula:

(1) $$\begin{array}{ccc} & & \mathrm{Encapsulation}\;\mathrm{efficiency}\;\mathrm{of}\;\mathrm{TTob}\left(\%\right)\\ & & \;=\left(\mathrm{Initial}\;\mathrm{mass}\;\mathrm{of}\;\mathrm{Tob}-\mathrm{Residual}\;\mathrm{mass}\;\mathrm{of}\;\mathrm{Tob}\right)/\\ & & \;\left(\mathrm{Initial}\;\mathrm{mass}\;\mathrm{of}\;\mathrm{Tob}*100\%\right) \end{array}$$

(2) $$\begin{array}{ccc} & & \mathrm{Loading}\;\mathrm{efficiency}\;\mathrm{of}\;\mathrm{TTob}\left(\%\right)\\ & & \;=\left(\mathrm{Initial}\;\mathrm{mass}\;\mathrm{of}\;\mathrm{Tob}-\mathrm{Residual}\;\mathrm{mass}\;\mathrm{of}\;\mathrm{Tob}\right)/\\ & & \;\left(\mathrm{Total}\;\mathrm{mass}\;\mathrm{of}\;\mathrm{TTob}*100\%\right) \end{array}$$

### Platelet Isolation and Platelet Membrane Derivation

To remove any remaining blood cells, the platelet‐rich plasma (PRP) obtained was subjected to centrifugation at 100*g* for 20 min. To minimize platelet activation, a modified 1 × PBS buffer supplemented with specific concentrations of EDTA (1 × 10^−3^ m, MedChemExpress, USA) and prostaglandin E1 (2 × 10^−6^ m, Macklin, China) was introduced into the purified PRP. Following this, the platelets were pelleted at room temperature by centrifugation at 800*g* for 20 min. The supernatant was carefully discarded, and the platelets were resuspended in 1 × PBS solution containing 1 × 10^−3^ m of EDTA. In addition, protease inhibitor tablets (MedChemExpress, USA) were added to the suspension for further stabilization. To obtain platelet membranes, a repeated freeze‐thaw procedure was used. Initially, small portions of the platelet suspensions were frozen at −80 °C and subsequently thawed at room temperature. The thawed suspensions were then subjected to centrifugation at 4000*g* for 3 min to pellet the platelet membranes. Following this, the pelleted platelet membranes underwent a series of three consecutive washes using a 1 × PBS solution containing protease inhibitor tablets. Finally, the pelleted platelet membranes were suspended in water, and sonication was performed for 5 min using a sonicator operating at a frequency of 42 kHz and a power of 100 W.

### Neutrophil Isolation and Neutrophil Membrane Derivation

To collect neutrophils from bone marrow, a modified Percoll gradient method was used using BALB/c mouse.^[^ ^42^ ^]^ To isolate bone marrow cells, the knee joint was dissected, and the femurs and tibias were separated. The marrow from two femurs and two tibias per mouse was flushed using a 5 mL syringe and a 25‐gauge needle, with a solution of 1 × HBSS and 0.38% sodium citrate buffer. The bone marrow was gently disaggregated by repeatedly aspirating and expressing the solution using an 18‐gauge needle and a 5 mL syringe, resulting in a single‐cell solution. The disaggregated bone marrow was then transferred to a clean 50 mL conical tube and centrifuged at room temperature, 800*g* for 5 min. After discarding the supernatant, the pellet was resuspended in 1 × HBSS with 0.38% sodium citrate buffer. Next, the cell suspension was layered on top of a Percoll gradient with a density of 1.077/1.090 g mL^−1^ (Servicebio, China). Centrifugation was carried out at 800*g* for 30 min. Neutrophils were collected from the interface between the 1.077 and 1.090 g mL^−1^ layers. Subsequently, the obtained sample was treated with RBC lysis buffer to lyse the erythrocytes. The neutrophils underwent three rounds of washing with 1 × PBS and were finally suspended in serum‐free RPMI medium. They were stored at −80 °C until further membrane derivation. The separation of neutrophils’ plasma membrane was achieved using a previously established technique.^[^ ^43^ ^]^ Thawed frozen cells underwent three rounds of washing with 1 × PBS through centrifugation at 800*g*. Subsequently, the cells were resuspended in a hypotonic lysing buffer containing a combination of protease and phosphatase inhibitors, along with 225 × 10^−3^ m d‐mannitol, 75 × 10^−3^ m sucrose, 0.2 × 10^−3^ m EGTA, and 30 × 10^−3^ m Tris‐HCl (pH 7.5). The chemicals used in this process were sourced from Macklin, China. To disrupt the cells, a Dounce homogenizer (Wheaton, USA) equipped with a tightly fitting pestle was used, with 20 passes performed. Following homogenization, the solution was centrifuged at 4 °C and 20 000*g* for 25 min. The resulting pellet was discarded, and the supernatant was subjected to a second centrifugation step at 4 °C and 100 000*g* for 35 min. The membranes were washed twice with 0.2 × 10^−3^ m EDTA in water. The pellet obtained from this centrifugation step contained the membranes. The membrane content was quantified using a BCA kit (Biosharp, China), with bovine serum albumin (BSA) serving as the standard. The membranes were suspended in 0.2 × 10^−3^ m EDTA and stored at −80 °C for subsequent experiments.

### Membrane Fusion and TTob Enveloping

Following the isolation of neutrophil and platelet membrane vesicles, the two membranes were fused together under five minutes of ultrasonication (42 kHz, 100 W). This process produced the hybrid membranes. Then, TTob@NPM was prepared by coextruding TTob and hybrid membranes through 400 and 200 nm polycarbonate filter membranes with a liposome extruder (Merck, USA).

### Nanoparticle Characterization

The hydrodynamic size and surface zeta potential of tFNAs, TTob, PV, NV, and TTob@NPM were determined using DLS (Nano ZS, Malvern, UK). To assess the nanoparticles’ morphology and size, each sample was carefully placed on carbon‐coated copper grids and allowed to settle for 5 min. Excess fluid was removed using filter paper, and the samples were then stained with 1% uranyl acetate for 5 min. Subsequently, TEM (HT7700, Hitachi, Japan) was utilized for visualization. For fluorescence imaging experiments, DiI (excitation = 549 nm/emission = 565 nm), DiO (excitation = 484 nm/emission = 501 nm), and Cy5 (excitation = 651 nm/emission = 670 nm) were encapsulated into NV, PV, and TTob, respectively. Fluorescence colocalization was then imaged using CLSM. To test the presence of membrane protein, SDS‐PAGE and western blotting analysis were performed. In the initial step, all samples were centrifuged at 10 000*g* for 5 min at 4 °C. The resulting deposits were lysed using a lysis buffer (KeyGEN, China). The lysates were mixed with 5 × loading buffer in a volume ratio of 4:1 and subsequently boiled at 100 °C for 5 min. Following that, the samples were separated on a polyacrylamide gel, and the gel was visualized using an enhanced chemiluminescence detection system (Bio‐Rad, USA) after staining with Coomassie blue (Biosharp, China). For the western blot analysis of cd36 (a marker for platelet membrane) and cd11b (a marker for neutrophil membrane), the gel was transferred onto a polyvinylidene difluoride membrane (TaKaRa, Japan). After a 15‐min incubation in rapid blocking buffer, the samples underwent overnight incubation at 4 °C with the primary antibodies (ab252922, 1:1000, Abcam, UK; ab133357, 1:1000, Abcam, UK). Subsequently, the secondary antibody (1:3000, Beyotime, China) was added, and the mixture was incubated for 1 h at room temperature. The final evaluation was performed using an improved chemiluminescence detection system (Bio‐Rad, USA). Stability analysis was conducted using PAGE after incubating the samples at various time points in 4 °C PBS, 37 °C *P. aeruginosa* supernatant, and 37 °C 10% FBS.

### Planktonic *P. aeruginosa* Antimicrobial Activity Studies

The PAO1 strain was acquired from frozen glycerol stocks and subjected to overnight culture in trypticase soy broth (TSB) medium at 37 °C and 220 rpm. To ensure optimal bacterial growth in the logarithmic phase, PAO1 cultures were 1:8 diluted into TSB medium and shaken for 3 h to reach a >0.4 optical density at 600 nm (OD600).^[^ ^44^ ^]^ Subsequently, PAO1 was seeded into 96‐well plates at 100 µL with an initial OD600 of 0.002. Treatment and control groups, each comprising 100 µL, were added to achieve a final OD600 of 0.001. The concentration of Tob in the treatment groups started at 128 × 10^−6^ m, with successive 1:2 serial dilutions until reaching a Tob concentration of 0.25 × 10^−6^ m. All antimicrobial experiments were performed with a minimum of three biological replicates (*n* = 3). The plates were then incubated at 37 °C and 220 rpm for 12 h, following which the OD600 was measured to evaluate the bacterial burden. The MIC values were determined as the lowest drug concentration that inhibited 98% of bacterial growth compared to the untreated controls.

### *Aeruginosa* Biofilm Antimicrobial Activity Studies

PAO1 biofilms were cultivated using established techniques.^[^ ^45^ ^]^ Overnight cultures of PAO1 were diluted 1:8 into TSB medium and further incubated for 1–3 h until an OD600 of >0.4 was reached, confirming bacterial growth in the logarithmic phase. Subsequently, the bacteria were seeded into 96‐well plates at 200 µL with an initial OD600 of 0.02 and incubated at 37 °C for 72 h, with the medium being refreshed every 24 h. After 72 h, the supernatant was discarded, and the treatment groups (200 µL per well) were introduced to the biofilms. The plates were then incubated at 37 °C for 12 h. Subsequently, live/dead bacterial dyes (Uelandy, China) NucGreen and EthD‐III were gently added dropwise to each well, ensuring no disturbance to the biofilm. CLSM was used to image the fluorescence of NucGreen (excitation = 503 nm/emission = 530 nm) and EthD‐III (excitation = 530 nm/emission = 620 nm) in bacterial biofilms. Then images were captured. Each image consisted of z‐stacks of the biofilms, with a spacing of 2 µm and a total of 70 slices spanning 70 µm. The z‐stacks were centered at the z‐position where the NucGreen fluorescence intensity was maximum. In addition, relative z‐stacks were generated by including slices ±35 µm from the center slice, thereby covering the entire biofilm. All biofilm experiments involved at least three biological replicates (*n* = 3). Bacterial vitality and biomass volume were quantified using ImageJ on biofilm z‐stacks. Before conducting the analysis, background subtraction was applied to all images in the stack. Following that, the average fluorescence intensities of NucGreen and EthD‐III were detected for each z slice. Furthermore, a ratio of EthD‐III to NucGreen fluorescence intensities was determined. To analyze biomass, crystal violet staining was utilized. The PAO1 biofilms underwent fixation with formalin for 1 h after various treatments for 12 h, followed by natural drying. Subsequently, staining was performed using a 0.02% crystal violet solution (Biosharp, China) for 30 min, followed by three saline washes. The PAO1 biofilms were then observed using an inverted microscope. For biofilm thickness analysis, the relevant parameter Biofilm_MeanThickness (the mean of the thickness of the biofilm at the x‐y‐position of the object) was extracted and calculated with BiofilmQ software tool.^[^ ^46^ ^]^

### Chemotaxis Experiments

Chemotaxis experiments were conducted using the µ‐Slide Chemotaxis chamber (Ibidi, Germany) to investigate TTob@NPM migration. TTob@NPM were pipetted into the seeding chamber, and the lower and upper chambers were filled with PBS containing or lacking *P. aeruginosa* overnight cultures (OD600 > 0.4), respectively. After allowing TTob@NPM to settle for 15 min, CLSM was used for examination. Micro‐images were captured at 15 s interval over a period of 2 h. The motion behavior of TTob@NPM was analyzed using ImageJ with tracking plugins.^[^ ^47^, ^48^ ^]^ There were several parameters that illustrate the chemotaxis of TTob@NPM, including:

1. Straight line distance: the distance between the last spot of the track and the first spot of the track in time.
2. Straight line velocity: the straight line distance divided by the time.
3. Accumulated distance: the full distance the particle traveled throughout the track.
4. Velocity: the accumulated distance divided by the time.
5. Directionality: the straight line distance divided by accumulated distance. It is a unitless value that ranges from 0 to 1. Values close to 0 suggest restricted movement, where the particle remains near its starting position, while values close to 1 indicate linear movement with a consistent direction.

### Biodistribution of TTob@NPM

Mice infected with *P. aeruginosa* were given Cy5‐labeled TTob@NPM intranasally to assess its in vivo biodistribution and effectiveness in targeting inflammation. The mice were euthanized after the administration was given at various time points (2, 4, and 6 h). The primary organs were then removed and examined using a whole‐body fluorescent system (IVIS Lumina III Series, PerkinElmer, USA). Lungs were preserved using an OCT embedding medium, frozen, and sectioned. These tissue sections were subsequently subjected to DAPI staining. The fluorescence images were captured using a slide scanner (VS200, Olympus, Japan).

### Assessment of TTob@NPM Penetration in EPS‐Mimic Gel

Initially, PAO1 biofilms were spread out in TSB (1 × 10^6^ CFUs mL^−1^) and incubated for 24 h at 37 °C in a six‐well microplate. The suspension was then centrifuged for 3 min at 12 000 rpm. Following the removal of the uppermost layer containing the culture media, the residual liquid was passed through a hydrophilic nylon filter with a pore size of 0.2 µm (Merck, USA). To create an EPS‐mimic gel, a 3 mL EPS dispersion was then resuspended and supplemented with 1.5 wt% agarose. Then, to the top of the EPS‐mimic gel, 200 µL of the Cy5‐labeled TTob suspension and an equivalent quantity of Tob in the Cy5‐labeled TTob@NPM solution were added separately, and the gel was incubated for 12 h at 37 °C before fluorescence detection.

### Binding and Neutralization of Toxin

Using leucocidin (MedChemExpress, USA) as a model toxin, TTob@NPM was investigated as a toxin decoy to bind and neutralize PFTs. The TTob@NPM was combined with a fixed concentration of commercial leucocidin, and it was ultrasonically treated for 5 min. The combination was placed into a 5% pure RBC solution after the ultrasonic treatment, and it was then incubated at 37 °C for 1 h and 24 h. Following this incubation time, the 5% RBC solution was centrifuged, and the supernatant's absorbance was measured at 540 nm (the oxyhemoglobin absorbance spectra) to determine the extent of hemolysis.

### Drug Release from TTob@NPM

To test the Tob release rate, TTob@NPM solution was incubated with bacteria suspension at 37 °C for various time period (1, 3, and 24 h). The concentration of released Tob was assessed via an *o*‐phthalaldehyde fluorescence assay.^[^ ^49^ ^]^ In brief, *o*‐phthalaldehyde solution was prepared by dissolving it at a concentration of 1 mg mL^−1^ in 100 × 10^−3^ m Na borate buffer (pH = 10.4). Methanol and β‐mercaptoethanol were added to achieve final concentrations of 10% (v/v) and 0.5% (v/v), respectively. To prepare the analysis samples, 15 µL of *o*‐phthalaldehyde solution was mixed with 90 µL of isopropanol, followed by the addition of 15 µL of the sample supernatant. The solutions were incubated for 20 min before fluorescence intensity analysis was performed (excitation = 360 nm/emission = 450 nm). The obtained fluorescence intensities were compared to the Tob calibration curves.

### Binding of Bacteria

To evaluate the selective binding capability of TTob@NPM, PAO1 was utilized as a representative pathogen adhering to phospholipid surfaces. The bacteria were cultured overnight on TSB agar at 37 °C. A single colony was then inoculated into TSB medium and incubated at 37 °C with rotary shaking. The overnight culture was subsequently diluted 1:100 in fresh TSB medium and further incubated for 3 h with shaking. Then Cy5‐labeled TTob@NPM was combined with the NucGreen‐stained bacteria suspension (5 × 10^8^ CFUs mL^−1^) and incubated for 5 min under an ultrasonic field. Following the ultrasonic incubation, the fluorescence of Cy5 and NucGreen was detected using CLSM.

### SEM

SEM was used to examine the morphology of both the bacteria and TTob@NPM. For sample preparation, 20 µL droplets of various suspensions were placed onto carbon substrate‐coated SEM stubs and left to air dry. Subsequently, the samples were stained with UranyLess. To accomplish this, a droplet of UranyLess was applied to a hydrophobic surface, such as parafilm, and the stubs containing the samples were placed onto the droplet for a duration of 2 min. Any excess stain on the samples was then removed by blotting the stubs with filter paper. Following this, the samples were washed three times with ddH_2_O at room temperature. Prior to SEM analysis, the samples were allowed to air dry naturally for a minimum of 12 h at room temperature.

### Care and Use of Mice

Male BALB/c mice, aged six weeks, were procured from Ensiweier company (China). All animal experiments adhered to the Guidelines for the Care and Use of Laboratory Animals of Sichuan University and were approved by the Animal Ethics Committee of Sichuan University (approval number: WCHSIRB‐D‐2023‐655). The mice were housed under controlled conditions with a temperature of 22 °C ± 3 °C, humidity maintained at 60%–70%, and a 12‐h light/dark cycle.

### Safety Assessment

Prior to the in vivo use, the safety of several nanoparticles was evaluated. This evaluation included a hemolysis assay, blood cell counts, a thorough metabolic panel, histological examination of key organs, and cytokine analysis. Whole blood from healthy male BALB/c mice was obtained and placed in tubes with an anticoagulant solution for the hemolysis experiment. To separate RBCs, blood was centrifuged at room temperature for 15 min at 900 × *g*. The collected RBCs were gently washed with PBS. Subsequently, RBCs (1 × 10^9^ cells) were exposed to various chemicals and incubated at 37 °C for 2 h. Following the treatment, all samples were centrifuged for 15 min at 900 × *g*. Hemolysis was evaluated by measuring the absorbance of the supernatant at 540 nm using a microplate reader from Thermo Scientific (USA). For the assessment of complete blood counts and comprehensive metabolic panel, both serum and whole blood samples were collected. Laboratory tests were then performed on these samples. To conduct histological analysis, the major organs were subjected to H&E staining. To analyze cytokine levels, the BALF was centrifuged at 700 × *g* for 5 min to separate the supernatant. The concentrations of cytokines in the BALF were subsequently measured using a multiplexed sandwich enzyme‐linked immunosorbent assay kit from Mlbio (China).

### Acute *P. aeruginosa* Models of Lung Infection

Mouse models of lung infection were conducted following established techniques.^[^ ^50^ ^]^ PAO1 strain was cultured overnight in TSB medium at 37 °C and 220 rpm. On the day of infection, the PAO1 cultures were 1:8 diluted into TSB and incubated for 3 h to reach a >0.4 OD600. The mice were randomly divided into six groups: blank (healthy), control (*P. aeruginosa*‐infected), tFNAs, Tob, TTob, and TTob@NPM. Subsequently, the anesthetized mice were intratracheally administered 2 × 10^6^ CFUs of PAO1 per mice in a volume of 50 µL. One day after planktonic bacteria inoculation, Tob concentration in Tob, TTob, and TTob@NPM groups were given at dosages of 42.8 × 10^−6^ m, in a total injection volume of 50 µL and total injection mass of 1 µg. tFNAs concentration in tFNAs, TTob, and TTob@NPM groups were given at dosages of 0.1 × 10^−6^ m, in a total injection volume of 50 µL and total injection mass of 0.4 µg.

### Chronic *P. aeruginosa* Models of Lung Infection

To address chronic lung infection in mice, agar bead‐encapsulated bacteria was utilized following the methodology described in previous research.^[^ ^51^ ^]^ Specifically, agar beads containing 2 × 10^6^ CFUs of PAO1 were dispersed in 50 µL of PBS and administered intranasally to the mice. After 3 d of infection, the treatment agents were intranasally administered to the mice, following the same treatment approach as in the acute *P. aeruginosa* models. To evaluate the outcomes, we recorded the survival and weight of the mice, with the observers blinded to the experimental groups. At 9 d post‐treatment, we collected and fixed the lungs in formalin for histological examination. In addition, lung tissue was collected from the infected mice, and the bacterial count was enumerated using the standard plate‐counting method. The BALF samples were used to assess protein levels, TNF‐α levels, and IL‐6 levels. The levels of TNF‐α and IL‐6 were measured using enzyme‐linked immunosorbent assay kits (Mlbio, China) according to the manufacturer's instructions. Blood samples were used to assess white blood cell (WBC) numbers.

### Immunohistochemistry Staining

After harvesting the lung tissues, they were fixed and embedded. Slices of the lung tissue were then subjected to treatment with primary antibodies targeting IL‐6 (GB11117, 1:200, Servicebio, China). Following this, a secondary antibody was applied. The images were scanned using a slide scanner, and analyzed using ImageJ with the IHC profiler plugin.

### Immunofluorescence Staining

Following the treatment described earlier, the lung sections were fixed using 4% cold paraformaldehyde. Subsequently, the sections were permeabilized with 0.5% Triton X‐100 and blocked with 5% goat serum to prevent non‐specific binding. After washing with PBS, the samples were incubated overnight at 4 °C with primary antibodies against IL‐6 (GB11117, 1:200, Servicebio, China), IL‐10 (GB11534, 1:200, Servicebio, China), F4/80 (GB113373, 1:5000, Servicebio, China), CD11c (GB11059, 1:1000, Servicebio, China), CD206 (GB113497, 1:200, Servicebio, China), CD11b (GB11008, 1:3000, Servicebio, China), and Ly6G (GB11229, 1:300, Servicebio, China). On the following day, after further washing, the sections were incubated with the corresponding secondary antibody. In addition, the sections were stained with DAPI to visualize the cell nuclei. The stained lung tissue sections were then scanned using a slide scanner.

### Statistical Analysis

The results are reported as means ± standard deviation (SD). For comparisons between different groups and within the same group in each experiment, one‐ or two‐way analyses of variance (ANOVAs) were conducted. Tukey's post hoc test was used for further analysis. All statistical analyses were performed using Prism software. The threshold for statistical significance was set at *p* < 0.05.

## Conflict of Interest

The authors declare no conflict of interest.

## Supporting information



Supporting Information

Supplemental Video 1

## Data Availability

The data that support the findings of this study are available from the corresponding author upon reasonable request.
