# Supplementary material for: Hybrid Cell Membrane‐Engineered Nanocarrier for Triple‐Action Strategy to Address Pseudomonas aeruginosa Infection
Source: Adv Sci (Weinh). 2024 Dec 25;12(6):2411261. doi: 10.1002/advs.202411261 (PMC11809413; doi:10.1002/advs.202411261)
Supplement: Supplementary file 1 — Supporting Information [file ADVS-12-2411261-s001.docx]

Supporting Information

Hybrid Cell Membrane-Engineered Nanocarrier for Triple-Action Strategy to Address *Pseudomonas aeruginosa* Infection

Shunhao Zhang, Tianyu Chen, Weitong Lu, Yunfeng Lin, Mi Zhou^*^, Xiaoxiao Cai^*^

**Supplementary discussion**

For the mechanism driving TTob to disrupt biofilm, we attribute the improved antibacterial activity against biofilm to the biocompatibility and permeation ability of tFNAs. First, satisfactory bacteria endocytosis of tFNAs can be achieved through “corner attack”^1-3^. tFNAs can attack the bacteria surface and rotate themselves. Then the electrostatic repulsion of bacteria surface will be reduced and the uneven charge of tFNAs will be redistributed across the surface, embedding one of their corners to wrap and internalize. In addition, TTob remains a positive charge (62.5 ± 0.8 mV, Fig. 2N), which can autonomously permeate the bacteria surface with different surface charges. Second, apart from their penetration ability at cellular level, tFNAs also possess the capacity to penetrate whole tissues and bacteria biofilm^4-6^. The tissue penetration capacity of drug carriers is related to physical parameters, of which small size is a prerequisite for the excellent tissue permeability of tFNAs. As size increases, tissue permeability gradually decreases. TTob can maintain similar structure and size of tFNAs (Fig. 2M) to preserve the tissue penetration ability of tFNAs, which explains the TTob penetration in extracellular polymeric substances of biofilm. In addition, the incorporation of Tob into a tFNA structure protected the antibiotic, thus improving the efficiency of antibacterial activity.

For the mechanism driving nanocarrier motion, we attribute improved motion behavior to the advantages of cell membrane coating design. This design strategy preserves the structure and function of the cell membrane, particularly the specific functional proteins on the surface. First, given the great number of chemoattractant receptors sensing various signals and adhesion molecules for inflammation-induced counterreceptor binding in neutrophil membrane, TTob@NPM can target the infection site^7, 8^. This artificial chemotaxis-like targeting motion can be observed and rationalized as a stochastic process of “biased random walk” involving movement in all directions, interrupted by tumbling phases where no forward motion occurs, with the nanocarriers either rotating randomly or reorienting in response to a lack of attractant. When the attractant is present, the frequency of tumbling decreases, leading to an overall motion of the nanocarriers toward the attractant gradient. From this point of view, this phenomenon is also described as macroscopic chemotaxis or advanced chemokinesis than chemotaxis of an individual specimen^9-12^. Second, the incorporation of natural materials into synthetic materials provides a robust protection against immune response in physiological system. Disguising as autologous cells, TTob@NPM can eventually escape the phagocytosis of immune cells, which further improves the accumulation in infection site^13^. Third, the layer of natural cell membrane resists biofouling, thus ensuring lasting and improved motion following a chemotactic gradient in biological fluids^13^.

While cell membrane coating shows great potential for targeted drug delivery, several challenges still impact its efficiency. First, the processes of membrane extraction and coating require further refinement^7, 14^. For nanomaterials to inherit the functions of the cell membrane, achieving a complete and successful coating is essential. However, current protocols, including cell lysis, sonication, centrifugation, and extrusion, lead to some membrane loss at each stage. Additionally, ensuring membrane uniformity can be difficult. Another key challenge is ensuring the correct orientation of the cell membrane on the core to preserve its functionality. Therefore, there is a need to develop an integrated, automated system with effective quality controls to streamline the process on a single device. Second, since cell membranes contain various proteins and other bioactive substances responsible for the functions of membrane-coated nanomaterials, ensuring the long-term storage of these nanomaterials with intact bioactive membrane proteins is a key challenge that needs to be addressed^14^. Third, the off-target effects of cell membrane-coated nanomaterials must be carefully evaluated to avoid unintended accumulation in other tissues, which could result in drug resistance^7, 8^. The inflammatory microenvironment is a complex system involving pathogens, immune cells, cytokines, blood vessels, and the extracellular matrix. For effective drug delivery, it is crucial to account for these factors to ensure that therapeutic agents not only reach the infection tissues but also distribute effectively within the pathological areas. This requires a deep understanding of drug distribution, transport pathways, and the variables that influence them. As a result, cell membrane-based nanotherapeutics could be designed to adjust drug dosage based on feedback from the inflammatory microenvironment, combining diagnostic and therapeutic components within a smart nanorobot. This concept could be realized through real-time nanomaterials monitoring and a DNA origami-driven controlled drug release system^15^.

Some modifications can be made to further improve the targeting efficiency of TTob@NPM through controlling motion behavior at three levels: speed control, direction control, and cluster control^12^. First, for speed control, the current TTob@NPM design should be upgraded to a nanorobot system that can convert chemical or external energy into mechanical movement. For instance, immobilizing the urease asymmetrically onto the surface of TTob@NPM to construct a urease catalysis Janus nanorobot^16^. Urease catalyzes the breakdown of endogenous urea into ammonia and carbon dioxide, creating propulsion for the nanorobots. As the concentration of additional fuel increases, the speed of the nanorobots rises. Thus, regulating the fuel concentration significantly impacts the nanorobots' speed. Second, for direction control, the physical fields induced external forces should be designed. The movement of nanorobots can be directed by adjusting the intensity and orientation of the applied field^17^. For instance, incorporating magnetic metallic segments into TTob@NPM allows for magnetic propulsion and precise steering under the influence of an external magnetic field. Third, for cluster control, integration design of nanorobots with diverse structures or functionalities to create heterogeneous swarms is essential. As a general strategy for organizing disparate magnetic nanorobots proposed by Cao *et al*. has mentioned, leveraging the distinct magnetic properties of disparate magnetic nanorobots with elaborately regulated parameter sets of the rotating magnetic field can manipulate nanorobots to show transitions between synchronization and desynchronization, and eventually execute precise drug delivery^18^.

**
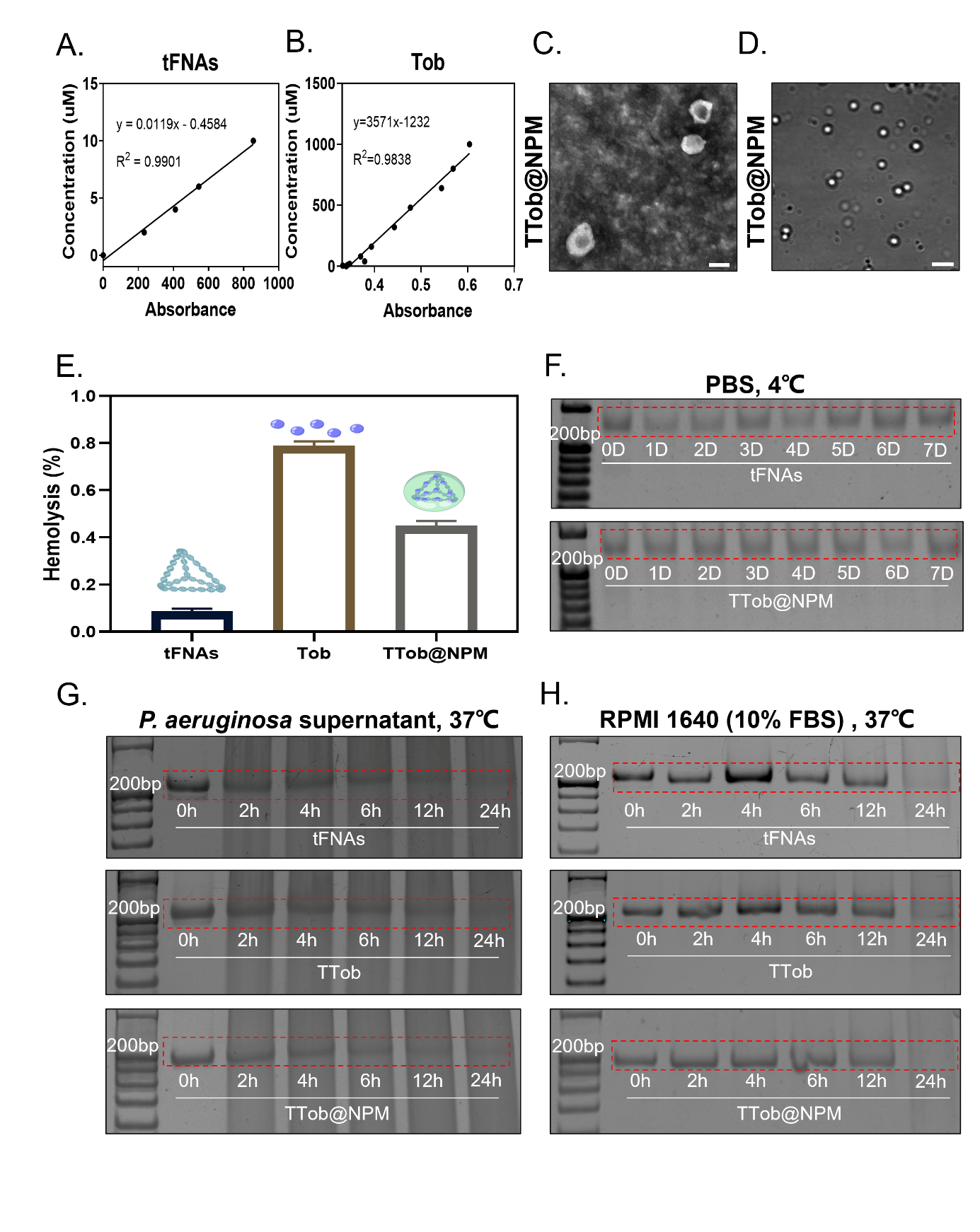
**

**Figure S1. Characterization of TTob@NPM.** **A)** Standard curve of tFNAs. **B)** Standard curve of Tob. **C)** The TEM images of TTob@NPM. Scale bar is 100nm. **D)** The CLSM images of TTob@NPM. Scale bar is 5μm. **E)** The hemolysis rate of tFNAs, Tob, and TTob@NPM. **F)** Representative PAGE images for stability of tFNAs and TTob@NPM in 4℃ PBS. **G)** Representative PAGE images for stability of tFNAs, TTob, and TTob@NPM in 37℃ *P. aeruginosa* supernatant. **H)** Representative PAGE images for stability of tFNAs, TTob, and TTob@NPM in 37℃ RPMI 1640 with 10% FBS. The error bars represented the SD. n ≥ 3. tFNAs, tetrahedral framework nucleic acids; Tob, tobramycin; TTob, tobramycin-loaded tFNAs complex; TTob@NPM, TTob encapsulated with neutrophil and platelet hybrid membranes.


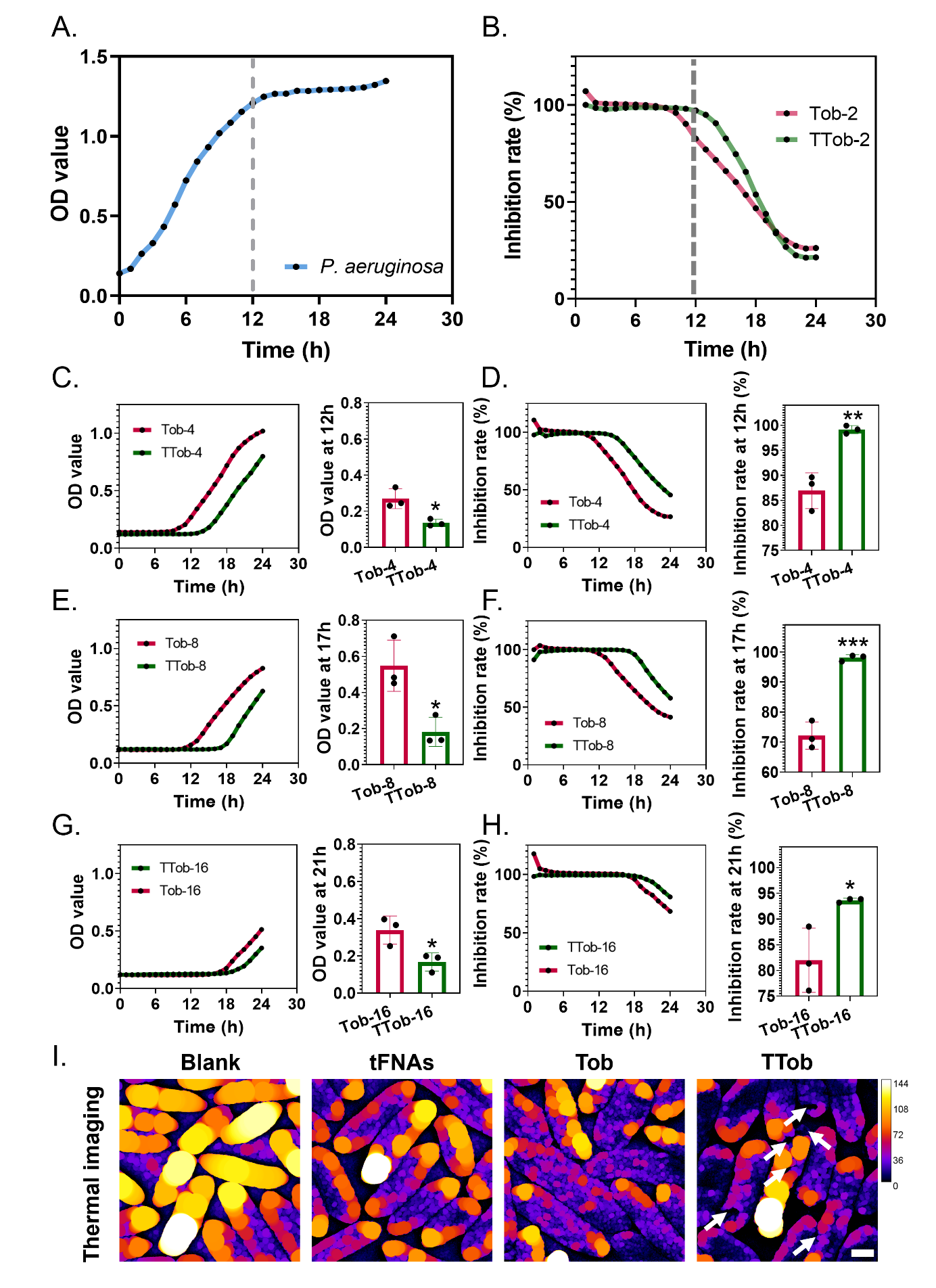


**Figure S2. Planktonic *P. aeruginosa* elimination capacity of Tob and TTob.**

**A)** The growth curve of *P. aeruginosa* across 24 hours. The dashed line indicates 12 hours, which is the end of logarithmic phase. **B)** The inhibition curves of *P. aeruginosa* with Tob-2 and TTob-2 treatments across 24 hours. The dashed line indicates 12 hours. The OD600 and inhibition rate of *P. aeruginosa* cultures were measured for 24 hours with Tob concentration at 4μM **(C-D)**, 8μM **(E-F)**, and 16μM **(G-H)**. **I)** Reconstruction of bacteria thickness using thermal imaging after different treatments. Scale bar is 500nm.The original SEM images of bacteria morphology are shown in Fig. 3E. The error bars represented the SD. ^*^ compared to the first group; ^*^ p < 0.05, ^**^ p < 0.01, ^***^ p < 0.001, and ^****^ p < 0.0001. n ≥ 3. Tob, tobramycin; TTob, tobramycin-loaded tFNAs complex.


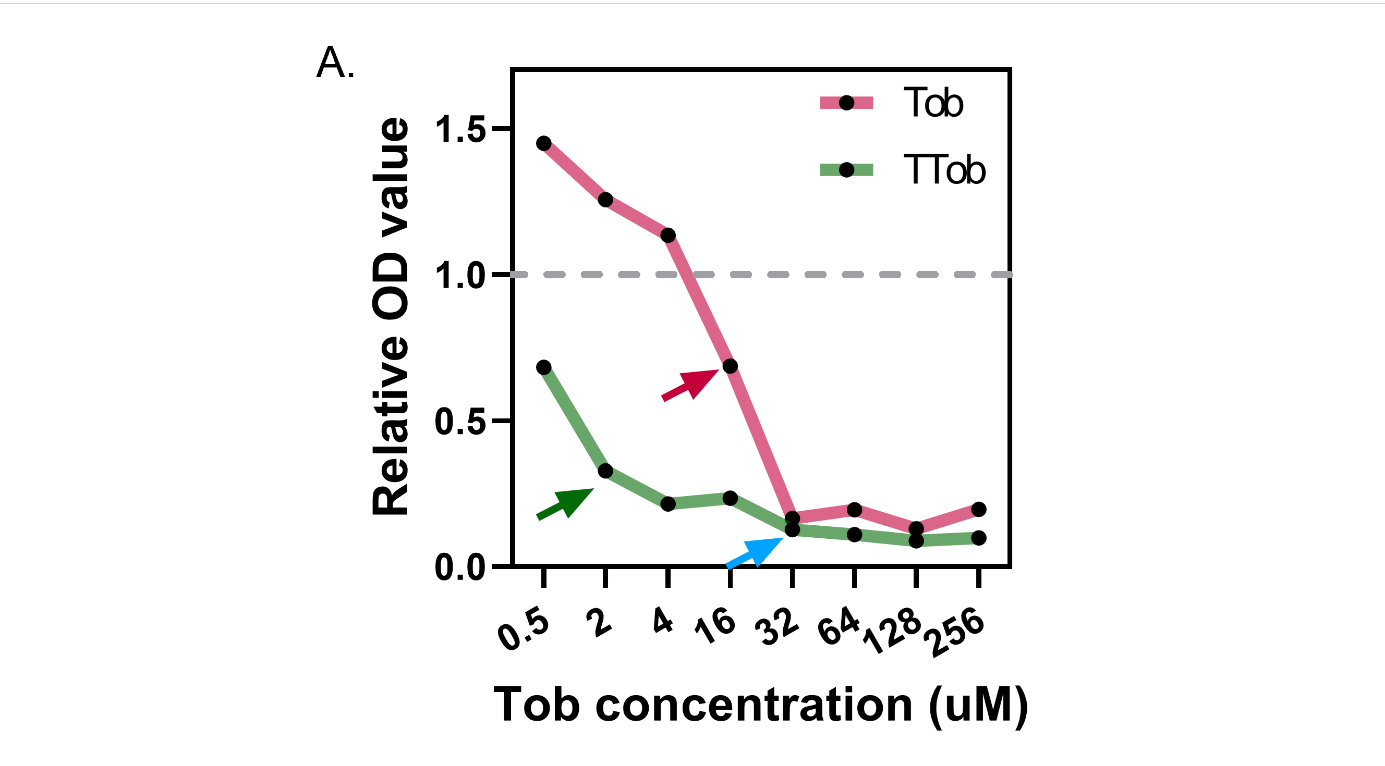


**Figure S3. Inhibition effect of Tob and TTob on *P. aeruginosa* biofilm growth.** **A)** Inhibition effect of Tob and TTob on *P. aeruginosa* biofilm growth with different Tob and TTob concentration at 12 hours. The dashed line indicates the base line. The green arrow indicates 2μM TTob, the red arrow indicates 16μM Tob, and the blue arrow indicates that TTob and Tob have comparable inhibition rate at 32μM. Tob, tobramycin; TTob, tobramycin-loaded tFNAs complex.


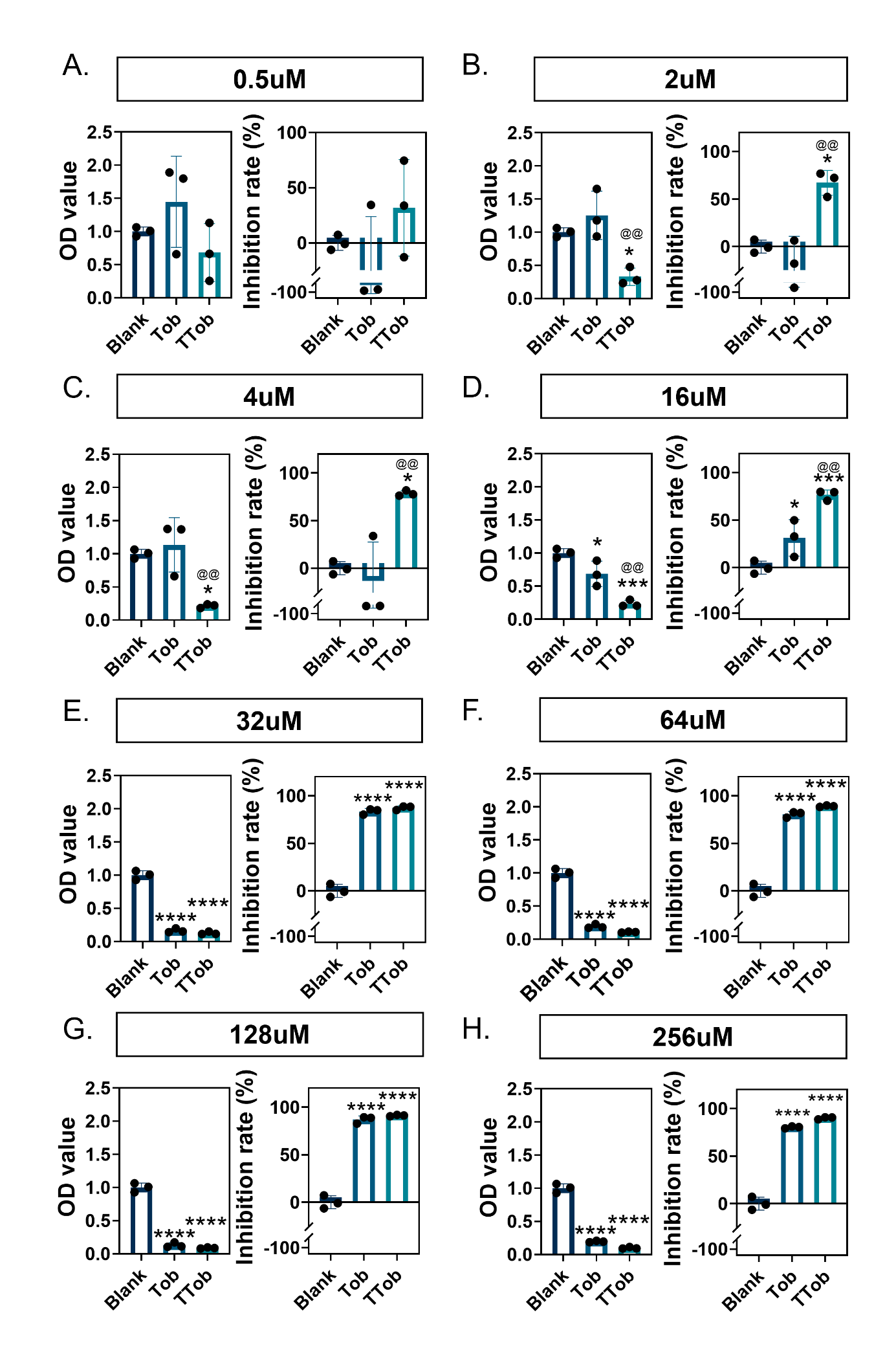


**Figure S4. *P. aeruginosa* biofilm elimination capacity of Tob and TTob.** The OD600 and inhibition rate of *P. aeruginosa* biofilm were measured with Tob concentration at 0.5μM **(A)**, 2μM **(B)**, 4μM **(C)**, 16μM **(D)**, 32μM **(E)**, 64μM **(F)**, 128μM **(G)**, and 256μM **(H)**. The error bars represented the SD. ^*^ compared to the first group; ^*^ p < 0.05, ^**^ p < 0.01, ^***^ p < 0.001, and ^****^ p < 0.0001. ^@^ compared to the second group; ^@^ p < 0.05, ^@@^ p < 0.01, ^@@@^ p < 0.001, and ^@@@@^ p < 0.0001. n ≥ 3. Blank, TSB medium; Tob, tobramycin; TTob, tobramycin-loaded tFNAs complex.


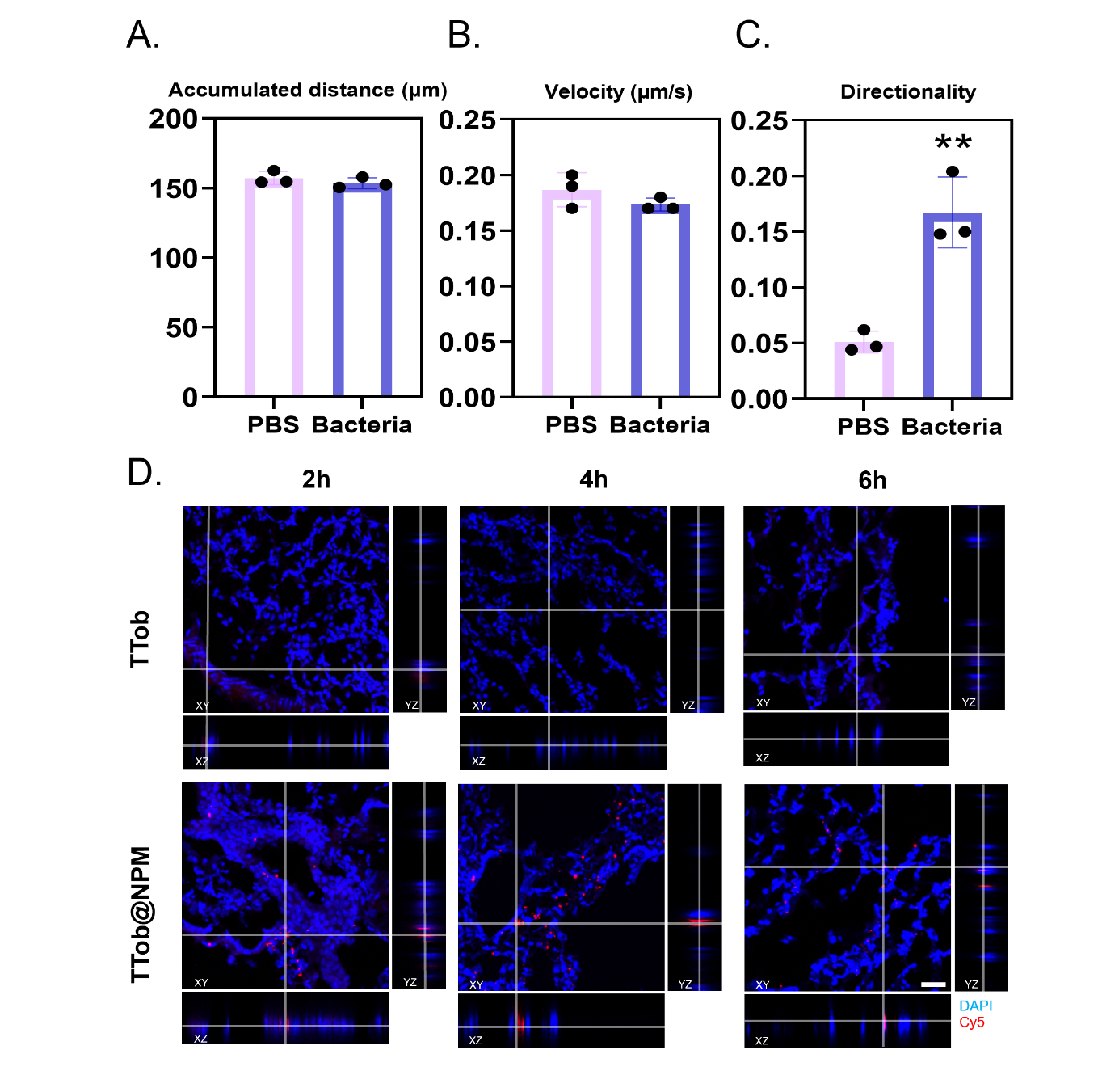


**Figure S5. Migration behavior *in vitro* and lung distribution *in vivo* of TTob@NPM.** **A)** Accumulated distance, defined as the full distance the particle traveled throughout the track, of TTob@NPM towards PBS and bacteria. **B)** Velocity, the accumulated distance divided by the time difference, of TTob@NPM towards PBS and bacteria. **C)** Directionality, the straight line distance divided by accumulated distance, of TTob@NPM towards PBS and bacteria. **D)** Lung distribution of TTob@NPM and TTob from xy, yz, and xz view. Red fluorescence (Cy5) indicates TTob@NPM or TTob. Blue fluorescence (DAPI) indicates nuclei. Scale bar is 25μm. TTob, tobramycin-loaded tFNAs complex; TTob@NPM, TTob encapsulated with neutrophil and platelet hybrid membranes.


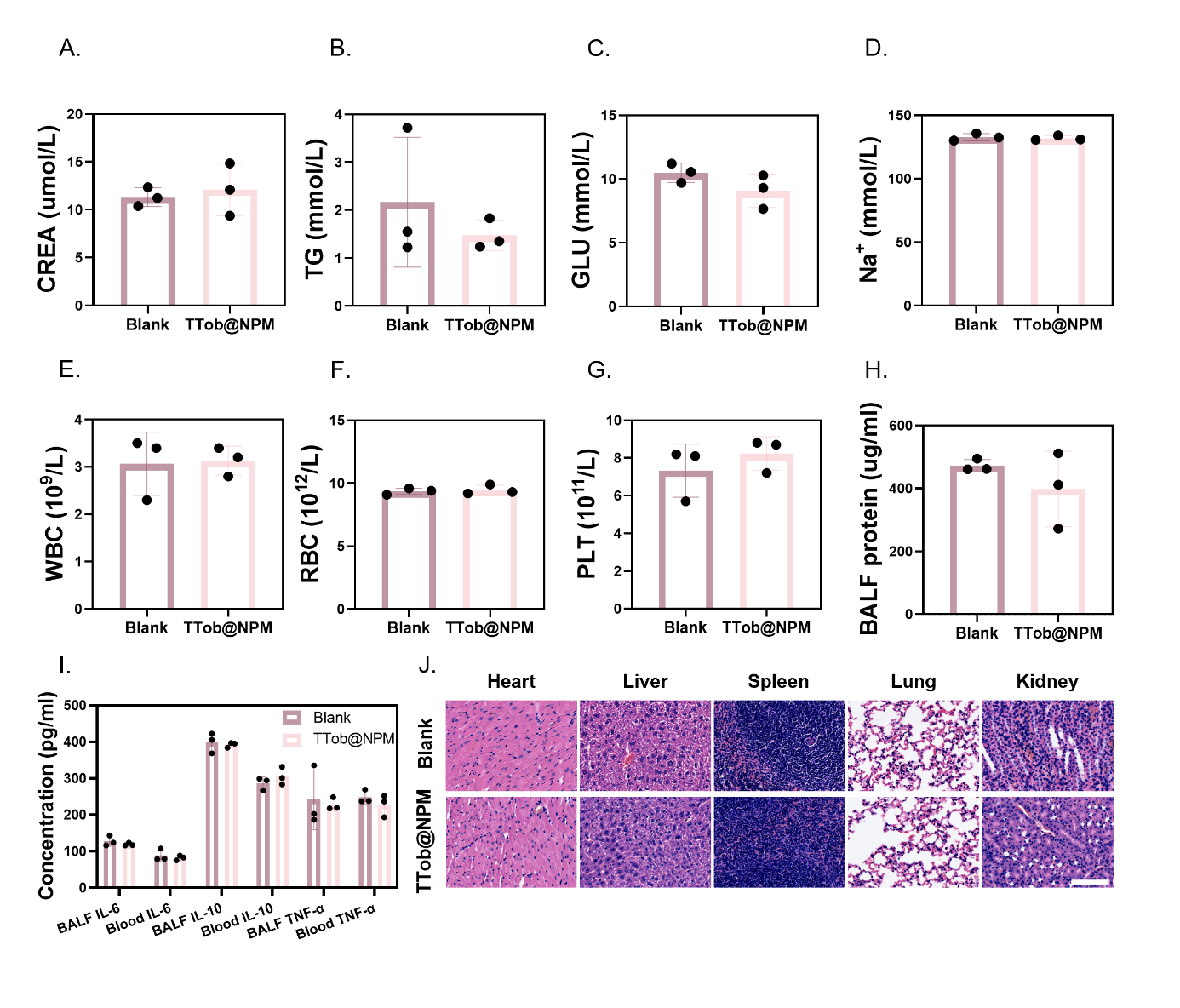


**Figure S6. Safety assessment of TTob@NPM.** Blood creatinine (CREA) **(A)**, triglycerides (TG) **(B)**, glucose (GLU) **(C)**, Na^+^ **(D)**, white blood cell (WBC) **(E)**, red blood cell (RBC) **(F)**, platelet (PLT) **(G)**, and bronchoalveolar lavage fluid (BALF) protein **(H)** levels after TTob@NPM administration. **I)** Cytokine levels after TTob@NPM administration. **J)** Representative images for H&E staining of major organs after TTob@NPM administration. Scale bar is 100μm. Blank, healthy group; TTob@NPM, TTob encapsulated with neutrophil and platelet hybrid membranes.


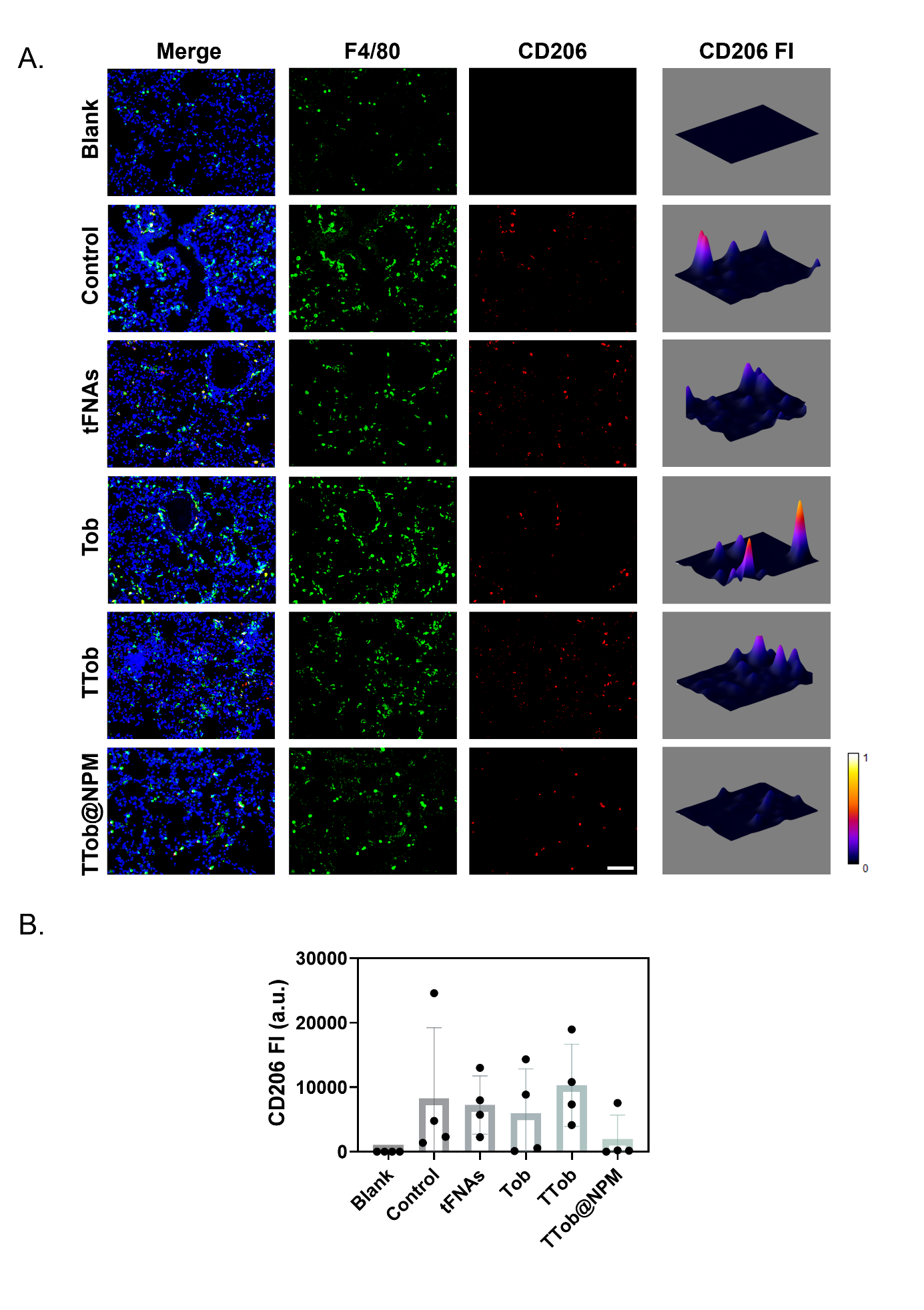


**Figure S7. Immunofluorescence analysis of M2-like macrophages levels in *P. aeruginosa*-induced acute lung infection *in vivo*.** **A)** Immunofluorescence staining of F4/80 (green), CD206 (red), and DAPI (blue) expression. Co-localization of the three signals indicate M2-like macrophages. FI, fluorescence intensity. Scale bar is 100μm. **B)** Quantitative analysis for immunofluorescence staining of M2-like macrophages level. The error bars represented the SD. n ≥ 3. Blank, healthy group; Control, infected group; tFNAs, tetrahedral framework nucleic acids; Tob, tobramycin; TTob, tobramycin-loaded tFNAs complex; TTob@NPM, TTob encapsulated with neutrophil and platelet hybrid membranes.


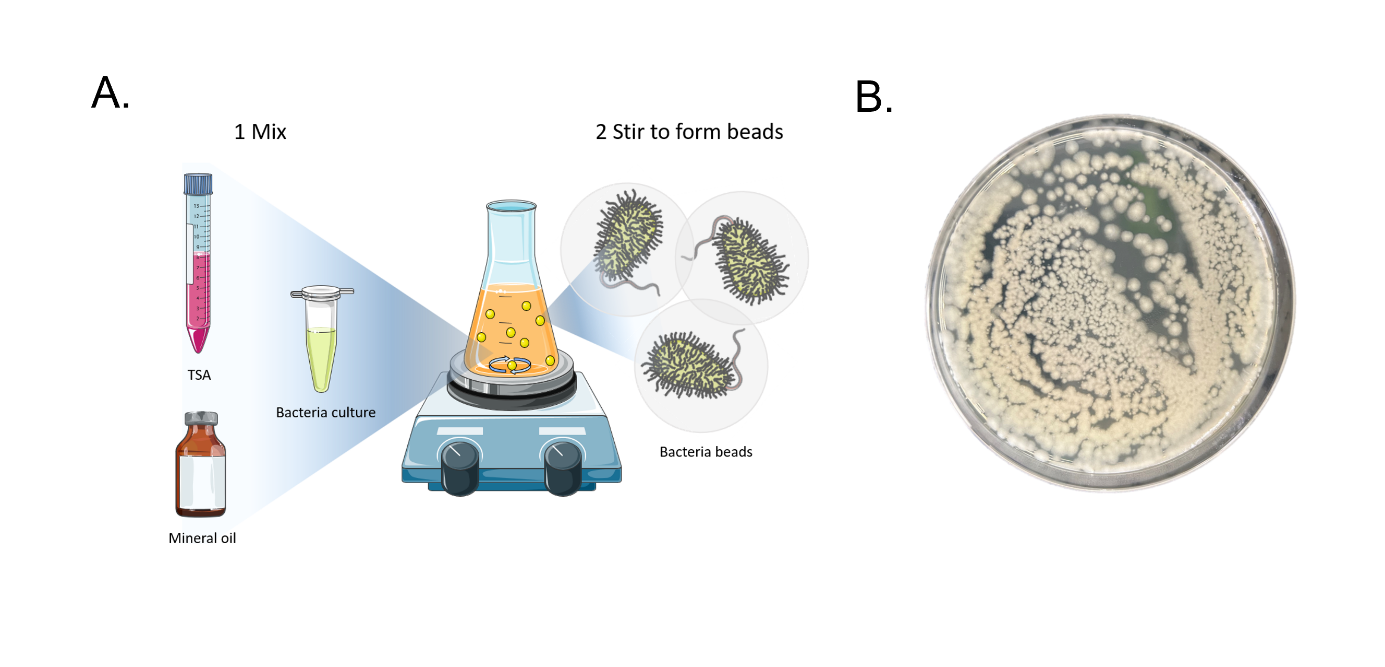


**Figure S8. *P. aeruginosa*-induced chronic lung infection model.** **A)** Schematic illustration for the process of the preparation of bacteria beads. **B)** A representative image of bacteria beads growing in an agar plate.


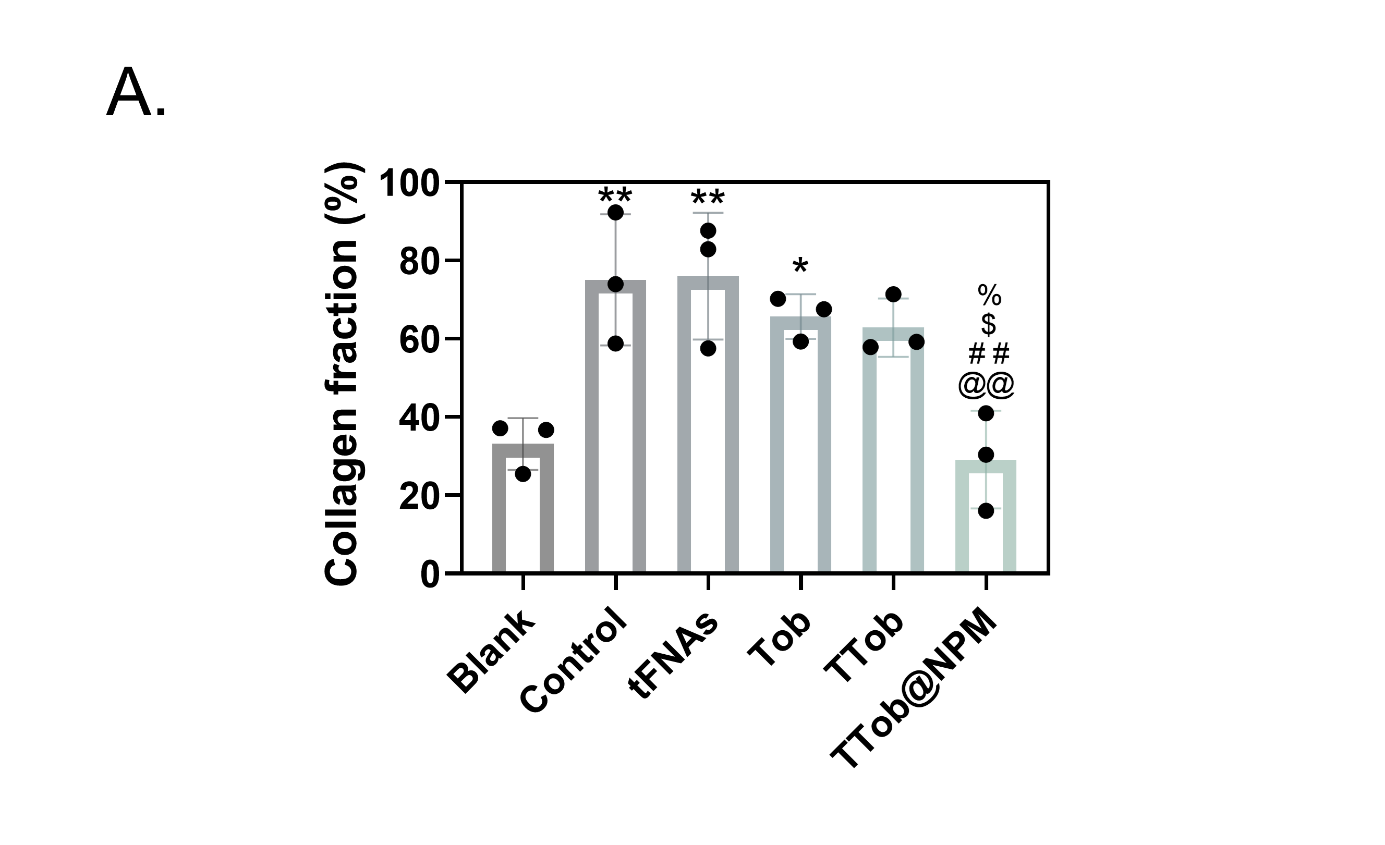


**Figure S9. Quantitative analysis for Masson’s trichrome staining in *P. aeruginosa*-induced chronic lung infection *in vivo*.** **A)** Quantitative analysis for Masson’s trichrome staining of chronic infected lungs after different treatments according to Figure 7O. The error bars represented the SD. ^*^ compared to the first group; ^*^ p < 0.05, ^**^ p < 0.01, ^***^ p < 0.001, and ^****^ p < 0.0001. ^@^ compared to the second group; ^@^ p < 0.05, ^@@^ p < 0.01, ^@@@^ p < 0.001, and ^@@@@^ p < 0.0001. ^#^ compared to the third group; ^#^ p < 0.05, ^##^ p < 0.01, ^###^ p < 0.001, and ^####^ p < 0.0001. ^$^ compared to the fourth group; ^$^ p < 0.05, ^$$^ p < 0.01, ^$$$^ p < 0.001, and ^$$$$^ p < 0.0001. ^%^ compared to the fifth group; ^%^ p < 0.05, ^%%^ p < 0.01, ^%%%^ p < 0.001, and ^%%%%^ p < 0.0001. n ≥ 3. Blank, healthy group; Control, infected group; tFNAs, tetrahedral framework nucleic acids; Tob, tobramycin; TTob, tobramycin-loaded tFNAs complex; TTob@NPM, TTob encapsulated with neutrophil and platelet hybrid membranes.

| ssDNA | Base sequence | Direction |
| --- | --- | --- |
| S1 | ATTTATCACCCGCCATAGTAGACGTATCACCAGGCAGTTGAGACGAACATTCCTAAGTCTGAA | 5’→3’ |
| S2 | ACATGCGAGGGTCCAATACCGACGATTACAGCTTGCTACACGATTCAGACTTAGGAATGTTCG | 5’→3’ |
| S3 | ACTACTATGGCGGGTGATAAAACGTGTAGCAAGCTGTAATCGACGGGAAGAGCATGCCCATCC | 5’→3’ |
| S4 | ACGGTATTGGACCCTCGCATGACTCAACTGCCTGGTGATACGAGGATGGGCATGCTCTTCCCG | 5’→3’ |

**Table S1.** The sequences for S1, S2, S3, and S4.

**Video S1. TTob@NPM migrated towards higher bacteria gradient.** The lower well was filled with overnight cultured planktonic bacteria (OD600 >0.4). Color lines indicated representative optical trajectories that were longer than 150 μm. Purple circles indicated nanocarriers. The directional motion of TTob@NPM from upper side to lower side with tumbling phases was observed.

**References**

1. Lin, Y. et al. Advances in regenerative medicine applications of tetrahedral framework nucleic acid-based nanomaterials: an expert consensus recommendation. *Int J Oral Sci* **14**, 51 (2022).

2. Wang, Q. et al. Modulation of Cerebrospinal Fluid Dysregulation via a SPAK and OSR1 Targeted Framework Nucleic Acid in Hydrocephalus. *Adv Sci (Weinh)* **11**, e2306622 (2024).

3. Li, Y. et al. A DNA tetrahedron-based ferroptosis-suppressing nanoparticle: superior delivery of curcumin and alleviation of diabetic osteoporosis. *Bone Res* **12**, 14 (2024).

4. Wiraja, C. et al. Framework nucleic acids as programmable carrier for transdermal drug delivery. *Nat Commun* **10**, 1147 (2019).

5. Li, S. et al. A Tetrahedral Framework DNA-Based Bioswitchable miRNA Inhibitor Delivery System: Application to Skin Anti-Aging. *Adv Mater* **34**, e2204287 (2022).

6. Chen, Y. et al. DNA framework signal amplification platform-based high-throughput systemic immune monitoring. *Signal Transduct Target Ther* **9**, 28 (2024).

7. Yan, H. et al. Engineering Cell Membrane-Based Nanotherapeutics to Target Inflammation. *Adv Sci (Weinh)* **6**, 1900605 (2019).

8. Liao, J. et al. Revolutionizing Neurocare: Biomimetic Nanodelivery Via Cell Membranes. *Adv Mater* **36**, e2402445 (2024).

9. Mou, F. et al. ZnO-based micromotors fueled by CO(2): the first example of self-reorientation-induced biomimetic chemotaxis. *Natl Sci Rev* **8**, nwab066 (2021).

10. Joseph, A. et al. Chemotactic synthetic vesicles: Design and applications in blood-brain barrier crossing. *Sci Adv* **3**, e1700362 (2017).

11. Plutnar, J. & Pumera, M. Chemotactic Micro- and Nanodevices. *Angew Chem Int Ed Engl* **58**, 2190-2196 (2019).

12. Tu, Y., Peng, F. & Wilson, D.A. Motion Manipulation of Micro- and Nanomotors. *Adv Mater* **29** (2017).

13. Li, J. et al. Biomimetic Platelet-Camouflaged Nanorobots for Binding and Isolation of Biological Threats. *Adv Mater* **30** (2018).

14. Liu, W.-L. et al. Recent Advances of Cell Membrane-Coated Nanomaterials for Biomedical Applications. *Adv Funct Mater* **30** (2020).

15. Li, L. et al. A DNA origami device spatially controls CD95 signalling to induce immune tolerance in rheumatoid arthritis. *Nat Mater* **23**, 993-1001 (2024).

16. Zheng, J., Qi, R., Dai, C., Li, G. & Sang, M. Enzyme Catalysis Biomotor Engineering of Neutrophils for Nanodrug Delivery and Cell-Based Thrombolytic Therapy. *ACS Nano* **16**, 2330-2344 (2022).

17. Si, L. et al. Swarming Magnetic Fe(3)O(4)@Polydopamine-Tannic Acid Nanorobots: Integrating Antibiotic-Free Superficial Photothermal and Deep Chemical Strategies for Targeted Bacterial Elimination. *Research (Wash D C)* **7**, 0438 (2024).

18. Cao, C. et al. Harnessing Disparities in Magnetic Microswarms: From Construction to Collaborative Tasks. *Adv Sci (Weinh)* **11**, e2401711 (2024).
